# Unveiling the Associations between EEG Indices and Cognitive Deficits in Schizophrenia-Spectrum Disorders: A Systematic Review

**DOI:** 10.3390/diagnostics12092193

**Published:** 2022-09-09

**Authors:** Andrea Perrottelli, Giulia Maria Giordano, Francesco Brando, Luigi Giuliani, Pasquale Pezzella, Armida Mucci, Silvana Galderisi

**Affiliations:** Department of Psychiatry, University of Campania “Luigi Vanvitelli”, Largo Madonna delle Grazie 1, 80138 Naples, Italy

**Keywords:** electroencephalogram (EEG), cognition, schizophrenia, biomarkers

## Abstract

Cognitive dysfunctions represent a core feature of schizophrenia-spectrum disorders due to their presence throughout different illness stages and their impact on functioning. Abnormalities in electrophysiology (EEG) measures are highly related to these impairments, but the use of EEG indices in clinical practice is still limited. A systematic review of articles using Pubmed, Scopus and PsychINFO was undertaken in November 2021 to provide an overview of the relationships between EEG indices and cognitive impairment in schizophrenia-spectrum disorders. Out of 2433 screened records, 135 studies were included in a qualitative review. Although the results were heterogeneous, some significant correlations were identified. In particular, abnormalities in alpha, theta and gamma activity, as well as in MMN and P300, were associated with impairments in cognitive domains such as attention, working memory, visual and verbal learning and executive functioning during at-risk mental states, early and chronic stages of schizophrenia-spectrum disorders. The review suggests that machine learning approaches together with a careful selection of validated EEG and cognitive indices and characterization of clinical phenotypes might contribute to increase the use of EEG-based measures in clinical settings.

## 1. Introduction

Cognitive dysfunctions are now widely recognized as pivotal features of schizophrenia-spectrum disorders. In recent decades, the interest in the study of cognitive deficits has grown, since they impact the daily functioning of subjects with schizophrenia more than other psychopathological dimensions and do not respond satisfactorily to current available treatments [1,2,3,4,5,6,7,8,9,10,11,12,13,14,15,16,17,18,19,20,21,22,23,24,25,26].

These deficits are present since the first manifestations of the disease when the first episode of psychosis (FEP) or of schizophrenia (FES) occurs [6,12,27], in subjects at clinical high risk of psychosis [28,29,30,31,32], as well as, in an attenuated form, in non-affected relatives of subjects with schizophrenia [17].

Most subjects with schizophrenia experience a broad range of cognitive deficits in different neurocognitive domains, such as working memory, attention/vigilance, verbal/visual learning, reasoning/problem solving and executive functioning [3,14,33,34,35,36]. In addition, subjects with schizophrenia often show impairments in social cognition, a cognitive domain defined as a range of abilities guiding the interpretation of other’s emotions or intentions, leading to informed conclusions and behaviours [1,2,4,16,25,37,38,39,40,41,42,43].

Throughout the years, several test batteries and assessment scales, such as the MATRICS Consensus Cognitive Battery (MCCB) [35,44,45,46] and the Brief Assessment of Cognition (BACS) [47], have been developed to provide a standardized evaluation of cognitive deficits in schizophrenia [48]. In addition to neuropsychological batteries and tests, neuroimaging studies have investigated these deficits and tried to untangle the associations between neuronal abnormalities and cognitive impairments in schizophrenia-spectrum disorders. For instance, studies employing functional magnetic resonance imaging (fMRI) have highlighted that neuronal abnormalities localized in temporal and frontal brain regions (temporal gyrus and the dorsolateral prefrontal cortex), as well as dysfunctions within broad neuronal circuits, such as frontal, striatal, parietal and thalamic circuits, could be at the core of the cognitive deficits observed in schizophrenia-spectrum disorders [49,50,51,52,53,54,55].

Numerous studies have also shown the presence of abnormalities in electroencephalographic (EEG) indices when subjects with schizophrenia-spectrum disorders and healthy controls were compared [56,57,58,59,60,61,62,63,64,65,66,67,68,69,70,71,72]. EEG recordings, due to their high temporal resolution, have been vastly employed to characterize the complex cascade of neuronal signalling underlying cognitive processing and to detect which steps of this processing might be impaired in subjects with severe mental health disorders [57]. Two main approaches have been used to investigate the electrophysiological correlates of cognition: the analysis of activity or connectivity in different EEG frequency bands and the analysis of event-related potentials (ERPs).

Frequency band analysis dissects EEG activity in its subcomponent frequencies, recorded either while subjects are at rest or while they perform a task. Different indices can be employed, such as the spectral power of spontaneous or evoked activity or parameters evaluating brain connectivity, which refers to the synchronization of neuronal signals across different regions of the cerebral cortex [73].

ERPs are wave deflections, time-locked to the occurrence of specific events of interest, such as the onset of visual or auditory stimuli or subjects’ responses during behavioural tasks [74]. Therefore, ERPs have been vastly employed to dissect the steps of the neural processing cascade supporting cognitive functions [75]. Finally, in addition to these two main types of EEG analysis approaches (frequency bands and ERPs), the accumulating evidence of sleep’s involvement in cognition has propelled the use of sleep-related measures, such as sleep spindles and K-complexes, for research purposes in neuroscience [76,77].

Nevertheless, although the use of EEG-based measures has allowed significant advances in understanding the neurobiology of cognitive functions, it has not yet led to the identification of reliable biomarkers for schizophrenia. Previous reviews, aimed to summarize results from electrophysiological studies, have focused mainly on the differences in EEG indices between patients and healthy controls, but have not provided an overview on the associations between these measures and cognitive dysfunctions in schizophrenia-spectrum disorders [56,58,78,79,80].

Enhancing knowledge on the neuronal bases of dysfunctions in different cognitive domains represents an important step, since it could help in the development of new effective treatments.

Therefore, this systematic review aims to provide a detailed report on the available evidence related to the associations between EEG indices and cognitive dysfunctions in schizophrenia-spectrum disorders.

## 2. Methods

### 2.1. Aim and Design of the Review

The present manuscript aims to provide a systematic review of studies focusing on correlations between EEG-based measures and cognitive domains, in order to discuss which electrophysiological indices might be used in clinical and research practice as potential biomarker of cognitive dysfunctions. The current systematic review search was performed in line with the PRISMA-Statement [81]. The studies selected included recordings during resting states or sensory and cognitive tasks in subjects with clinical and ultra-high risk of psychosis (CHR and UHR), first-episode psychosis (FEP) and first-episode schizophrenia (FES) or in subjects with a diagnosis of schizophrenia-spectrum disorders (SCZ). The choice of including subjects at-risk or who experienced a first episode of psychosis is motivated by the vast evidence of studies reporting cognitive deficits already during at-risk, prodromal and early phases of the illness [6,27,30].

### 2.2. Search Strategy

A systematic literature search was performed on 2 November 2021 with no time limit using the following databases: PubMed, Scopus and PsychInfo (Table 1). The keywords selected had to be included either in the title or in the abstract of the articles. In addition, reference lists were hand-searched to identify additional publications missed by the search strategy.

### 2.3. Selection Process and Eligibility Criteria

Any duplicate from the combination of the three databases was excluded. The remaining articles were included in the systematic review only if they met the following criteria:

*Inclusion criteria*

1. Meta-analyses, reviews, cohort and case–control articles published in English language and including human subjects;
2. Studies had to include data relevant to at least one EEG index, measured in subjects with at risk mental states, first episode psychosis or schizophrenia, or with a schizophrenia-spectrum disorder according to validated diagnostic criteria;
3. Studies had to include measurements of at least one cognitive domain using standardized tests or test batteries, or interviews.
4. Studies had to report at least one statistical analysis of correlation (Pearson’s or Spearman’s correlation) between one EEG index and a cognitive domain or a regression model in which an EEG index was used as a predictor of a cognitive domain.

*Exclusion criteria*

1. Book chapters, comments, editorials, case reports/case series, theses, proceedings, letters, short surveys, notes;
2. Studies irrelevant to the topic;
3. Full text unavailable.

Two researchers (A.P. and G.M.G.) independently screened for eligibility all the articles by titles and abstracts and then proceeded to read the full text. Discrepancies in the selection of the eligible articles have been discussed in advance with the whole group and were resolved by discussion and consensus.

### 2.4. Data Extraction

The following information was extracted onto customized sheets from included articles: authors and year of publication; domains of cognition considered; tests or scales employed to assess cognitive domains; EEG-based measures analysed in correlation with cognitive domains; comparison of the EEG indices between patients/at-risk samples and healthy controls; and outcomes of the analysis correlating electrophysiological data and cognitive scores. Three tables were generated according to the type of EEG index considered for the associations: frequency bands, ERPs and sleep EEG. Given the heterogeneity of the experimental paradigms, of the EEG indices and of the cognitive domains used in the eligible studies, we did not plan to carry out a meta-analysis.

## 3. Results

### 3.1. Characteristics of the Included Studies

**Figure 1:**
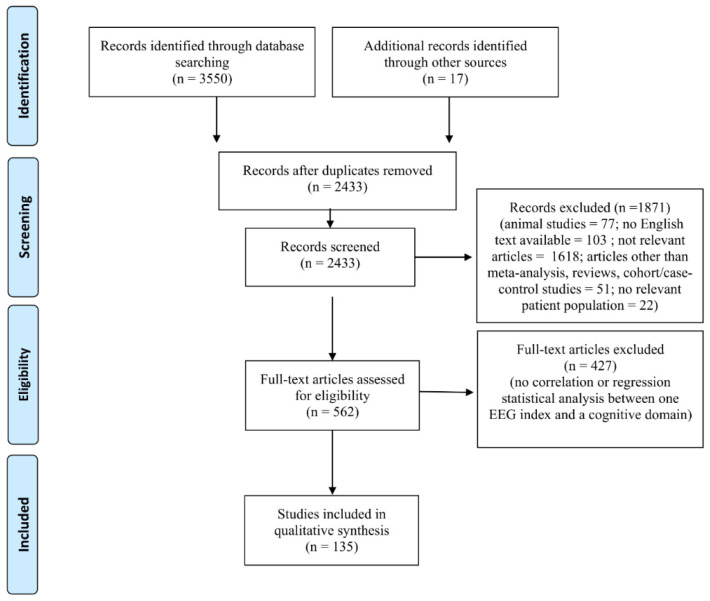
PRISMA flow chart of included studies. The PRISMA diagram details the search and selection process applied during our systematic literature search and review.

The combined outcome of the three databases results (Pubmed: 1733; SCOPUS: 460; PsycINFO: 1357) included 3550 records (Figure 1). In addition, 17 studies were included by hand search, yielding a total of 3567 studies. A total of 1134 studies were excluded because they were duplicates. After reading the titles and abstracts, 1871 were excluded since they did not meet the inclusion criteria (i.e., animal studies, no English text available, sample population that did not match the inclusion criteria) or were not relevant to the topic of the review. Four hundred and twenty-seven studies were eliminated because no data on associations between EEG indices and cognitive domains were found in the full text. Therefore, the final number of studies included was 135 (Figure 1).

### 3.2. EEG Frequency Bands Indices

Neuronal oscillations can be grouped in five main frequency bands, delta (0.5–4.0 Hz), theta (4–8 Hz), alpha (8–13 Hz), beta (13–30 Hz) and gamma (30–100 Hz) bands [57,82,83]. These data can be recorded either during a resting-state condition or during sensory stimulation and task performance. In addition to spectral power measures, other indices have been employed to investigate connectivity, synchronization and the level of neuronal activity integration across distributed cerebral networks.

The studies included in the following subsections are reported in Table 2.

#### 3.2.1. Delta and Theta Activity

Low frequency activity can be subdivided in delta (0.5–4.0 Hz) and theta (4–8 Hz) bands. Both bands appear to be involved in the orchestration of several cognitive processes such as working memory, detection of novelties, learning and allocation of attentive resources [124,125]. It has been reported that, in these two frequencies, subjects with schizophrenia present different abnormalities, often characterised by an increase in activity compared to physiological conditions [56,57,66,68,69,126,127,128].

Studies focusing on the relationship between delta activity and cognitive functions in schizophrenia-spectrum disorders are very few. In subjects with schizophrenia, delta studies reported an association between delta activity during resting-state conditions and emotion recognition [85], but found no association with attention, working memory, speed of processing, verbal and visual learning, reasoning or problem solving [86].

In task-related conditions, evoked delta activity in SCZ was positively (decreased EEG activity—worse cognitive performance) correlated with visual learning, attention, speed of processing [88] and social perception [87] and was negatively (increased EEG activity—worse cognitive performance) correlated with working memory [89]. Furthermore, delta activity coherence, as measured by intertrial coherence (ITC), showed a significant positive correlation with speed of processing [91]. However, other task-related studies [84,91] did not report an association between evoked delta activity and cognitive functions both in SCZ and CHR. Furthermore, Qu et al. [90] employed machine learning methods to develop a model combining clinical and electrophysiological variables (evoked activity in delta, theta and alpha bands and MMN amplitude) in order to predict cognitive impairments. In this study, the authors found that analysis of frequency band activity, including delta, did not contribute significantly to the model while only MMN did (as explained in Section 3.3.4).

A high number of studies focused on the association between theta activity and cognitive deficits. In SCZ three studies found significant negative correlations (increased EEG activity—worse cognitive performance) between theta power recorded at rest with cognitive functions, such as visuospatial memory [107], working memory [86], verbal learning [86,107], executive functioning [107] and emotion recognition [85]. In addition, theta band connectivity during resting state was a significant predictor of deficits in lexical processing [101] and verbal memory [92] in FES and CHR subjects and was also associated with deficits in the ability to initiate a consistent and coherent cognitive activity during a verbal fluency test in FES [102]. Finally, in FEP subjects, a positive correlation was found between executive functioning and verbal memory on the one hand and theta and gamma activity coupling at rest in the brain regions of the default-mode network on the other hand [103].

Many studies have also investigated task-related theta activity. In particular, in SCZ theta power evoked during working memory or visual tasks was correlated with working memory performance [89,98,99], attention [84], speed of processing [84,88,89], visual learning [88] and social perception [87]. Using a machine learning approach, Johannesen et al. [99], found that the machine learning model, combining the evoked activity of theta, alpha, beta and gamma bands, successfully predicted working memory performance. However, other studies did not find any significant correlation between theta power evoked during working memory or visual tasks with working memory [104,106], attention [106], and global cognitive scores derived from the BACS [106] or the MCCB [90] in SCZ and FEP.

Studies that analysed theta activity in SCZ during auditory paradigms found a significant correlation between social cognition and the theta band evoked power and phase locking values [96], and between verbal memory (but not working memory or conceptual flexibility) and theta amplitude [100]. In addition, no correlations were found between evoked theta power during an auditory oddball task and cognitive domains assessed with MCCB in FES and SCZ [108]. Interestingly, two studies focused on the effects of auditory-based targeted cognitive training (TCT) in schizophrenia and showed that higher baseline values of theta evoked power and phase-locking values could be used to predict greater improvements in attention, working memory and general cognitive abilities after the completion of the intervention [93,97]. Finally, theta connectivity during an auditory task was associated with processing speed, verbal fluency, verbal memory [95] and general cognitive abilities assessed with the BACS in SCZ subjects [94].

#### 3.2.2. Alpha Activity

Alpha activity is characterised by a frequency spectrum ranging from 8 to 13 Hz. Neuronal oscillations within this frequency band play a pivotal role in orchestrating cognitive functions, such as attention, working memory and cognitive control [129,130,131,132,133]. In subjects with SCZ most studies reported a decrease in absolute power during resting-state recordings [57,66,68,69] and disruptions in temporal coherence of evoked activity during sensory stimulation and cognitive tasks [134].

Studies focusing on associations between resting-state alpha features and cognition in SCZ have reported mixed results. Indeed, while some studies found a positive correlation (decreased EEG activity—worse cognitive performance) between alpha power and measures of working, visual and verbal memory [109] and emotion recognition [85], other studies did not report any significant correlation between this EEG measure and the six neurocognitive domains assessed through the MCCB [66], or verbal learning [86], working memory [86] and metacognitive functions [122]. Moreover, one study reported a positive correlation between individual alpha peak frequency (IAPF) and measures of speed of processing, perceptual reasoning, working memory, verbal reasoning and attention in SCZ [113]. Interestingly, one study investigating the effects of cognitive remediation found no significant correlation at baseline (pre-treatment) between IAPF and a global score of cognition but found that higher values in IAPF at baseline predicted higher responsiveness to the intervention and greater improvements on cognitive skills after the completion of the therapy sessions [111]. Finally, a decreased alpha band connectivity in the prefrontal cortex reported in FES as compared to controls was found to be correlated with cognitive processing impairments [101].

As to the evoked alpha activity, in SCZ a relationship between evoked alpha power and speed of processing [89] and working memory [99] has been detected. Furthermore, deficits in EEG signal coherence between cortical, temporal and occipital areas in the alpha band were related to worse vigilance skills in SCZ [112]. However, a lack of correlation between event-related alpha activity and different cognitive domains such as speed of processing, memory and learning [90,110], or between evoked alpha desynchronization and social cognition skills [87] have also been reported.

#### 3.2.3. Beta and Gamma

Beta (12–30 Hz) and gamma (30–100 Hz) frequency bands occupy the highest part of the neuronal activity spectrum. These bands have been found to be crucial for learning, top-down control, executive functions and formation of memories [135,136,137,138]. Dysfunctions in the activity and synchronization of beta and gamma bands have been vastly reported in schizophrenia [66,68,69,82,139,140]. However, clear-cut associations between beta activity and cognitive domains are not supported by the relevant literature. Two studies that used resting-state conditions in SCZ found that beta power was positively correlated (decreased in EEG activity—worse cognitive performance) with different cognitive functions, such as emotion recognition [85], vigilance, working memory, visual and verbal memory [109]. Furthermore, two studies investigating task-related activity reported a positive association of evoked beta bursts [114] and evoked beta activity [99] with working memory. However, also a lack of associations between beta power and verbal learning [86], working memory [86] and metacognitive functions [122] has been reported.

Very few resting-state studies investigated the associations between gamma band and cognition and reported discrepant results. Resting-state gamma power has been associated with the ability to consider multiple aspects of a situation (decentration) [122], with verbal learning [121] and working memory [121], but also a lack of association of this EEG index with working memory and verbal learning has been reported in SCZ [86]. In a study conducted in subjects with at risk mental state (ARMS) resting-state gamma activity was increased, as compared to HCs, and higher values were associated with better abstract reasoning [119].

Most of the studies focusing on gamma activity employed tasks or stimuli presentation. Two studies, using a memory task, found a negative correlation between gamma power and the performance on a working memory task in SCZ [98,99]. Another study, using an emotion perception task, showed that higher gamma synchrony elicited by facial expression stimuli was associated with higher scores of social cognition abilities (emotion identification, negativity bias and emotional intelligence) [123]. Studies employing an auditory task observed an association of gamma features with reasoning and problem solving, and with working memory skills in SCZ [117,120], as well as with working memory in FES [116]. Interestingly, one study focused on the effects of auditory-based targeted cognitive training (TCT) in schizophrenia, showing that higher baseline values of gamma evoked power could be used to predict greater improvements in general neurocognitive skills after the completion of the intervention [118]. In the context of studies relevant to gamma activity, recordings of auditory steady-state response (ASSR), an oscillatory brain response generated by the presentation of periodic auditory stimuli, have often been employed. One ASSR study found a positive correlation between gamma-band intertrial coherence (ITC) and event-related spectral perturbation (ERSP) indices and working memory skills in a large sample of SCZ [115]. However, other articles on ASSR data reported no significant associations between gamma activity and verbal memory [100,116,117], working memory [100], mental flexibility [100,117], attention [116,120], speed of processing [120], verbal [115,120] and visual learning [120] in SCZ and FES.

### 3.3. ERPs

The millisecond-level temporal resolution of ERPs has been precious to investigate the neuronal activity associated with processing of sounds and images and more complex cognitive processes such as the allocation of attentive resources and decision-making. ERPs represent an important tool for exploring the neurobiological bases of cognitive impairments in different disorders, such as ADHD [141], schizophrenia [56,142] and Alzheimer’s disease [143]. The studies and results reported in the following sections are described in Table 3.

#### 3.3.1. P50

The P50 is an early positive component of the auditory sensory responses, recorded 50 ms after a sound is presented. Generally, when two clicks are presented in a very brief interval, a reduction in P50 in response to the second click (S2) is observed, compared to the one produced after the first click (S1). This physiological process is known as sensory gating and can be analysed through the calculation of the P50 amplitude ratio (P50-S2/P50-S1) [58,144,147,148,149,150,151,152]. In subjects with SCZ, an increase in the P50 ratio is often reported, suggesting an impairment in sensory gating [58].

Studies evaluating the association between P50 ratio and cognitive functions in SCZ have reported inconsistent results, since some studies found a significant correlation between this EEG index and impairments in different cognitive domains such as attention [144,149,150], working memory [145,149], speed of processing [145], visual learning [146] and executive functions [150], while other studies found no significant correlations between P50 ratio and cognitive domains evaluated with the MCCB [151], verbal learning [144,145,147,148,150,152], memory [144,147,148,150,152], attention [145,147,148,152], processing speed [147,148] and executive functions [147,148].

#### 3.3.2. N100

The N100 is a large, negative-going evoked potential elicited by any unpredicted stimulus and related to the early stage of sensory processing [62]. Numerous studies assessing both N100 amplitude and sensory gating characteristics have found deficits in SCZ, which are thought to reflect an impairment in perceptual and attentional processing [62,228,229].

Deficits in N100 sensory gating were correlated to worse problem-solving abilities [154], attention [149] and working memory [149] in SCZ. However, the association between N100 amplitude and cognitive functions in SCZ is not clear since some studies reported a correlation between N100 amplitude and different cognitive abilities, such as verbal and visual memory [159,163], executive functioning [153], attention [201] and general cognition [153], while other studies did not find a significant correlation between N100 amplitude and attention [147,155,156], visual learning and memory [146,147,157,162], verbal learning and memory [160,161], executive functioning [147,161,162,197], working memory [164] or attention to stimuli related to social scenarios [158].

#### 3.3.3. P100

The P100 component is a positive potential, which peaks between 80 and 120 ms after stimulus onset. It primarily indexes perceptual stages of cortical visual processing, and its amplitude is influenced by selective attention [230,231,232]. In subjects with SCZ, compared with healthy subjects, reductions in P100 amplitude have frequently been reported in response to facial and non-facial stimuli [232,233].

Few studies have investigated the relationship between P100 and cognition, reporting inconsistent results: a positive correlation with attention deficits, suggesting an interference in effective sensory processing [155], or a negative correlation with the performance during a phonological task [165], or even a lack of association with the performance during a visuospatial attention [156] or a working memory task [164] have been reported in SCZ.

#### 3.3.4. MMN

The mismatch negativity (MMN) is a negative-going ERP component, which generally appears 150–250 ms after the presentation of unexpected stimuli [234,235,236,237]. The MMN reflects pre-attentive processing and is commonly regarded as an index of sensory memory [235,236]. This ERP is elicited through the auditory oddball paradigm and appears in response to the deviant tones, which are played rarely in a sequence of standard and frequent tones. The deviant tones can differ from the standard stimuli for different characteristics of the sound, such as frequency (eliciting, the pitch MMN, the pMMN), duration (eliciting dMMN), intensity, or for spatial location where the sound is played [238]. Abnormalities of both pMMN and dMMN have consistently been reported in patients with SCZ [79,239,240].

##### pMMN

In subjects with chronic SCZ, FES and CHR, deficits in pMMN amplitude have been associated with impairments in a variety of cognitive functions, such as verbal fluency [166], vigilance [192], verbal [174] and working memory [166,171], verbal learning [169], speed of processing [169], MCCB neurocognitive composite score [108,177] and emotion recognition [170,173]. However, some studies did not find associations between pMMN and cognitive composite scores [175] in SCZ, attention [197] in FEP subjects and verbal memory, verbal executive functions, spatial working memory, sustained attention and executive functioning in subjects with a prodromal syndrome of psychosis [168].

##### dMMN

Discrepant findings have also been reported for associations between dMMN amplitude and cognitive functions in SCZ, FEP and CHR. Most studies reported significant correlations between lower dMMN amplitude and impairments in several cognitive domains, such as reasoning [184], problem solving [184], verbal fluency [166,182], verbal learning [115,169,186,187], vigilance [192], visual attention [180,186,188], attentional switching [186], executive functioning [194], contextual processing [178], working memory [96,115,166,179,191,193], speed of processing [169,180,189], non-verbal memory [96], social perception [195], social cognition [96,181], abstraction and thought flexibility [96], emotion affective prosody [183,195], as well as composite cognitive scores [167,190]. An innovative longitudinal machine learning study [90] carried out in FEP subjects, used a combination of clinical, functional, cognitive and several EEG indices (delta, theta and alpha event-related activity and dMMN amplitude values recorded at multiple electrodes). The model showed that one group of subjects that presented an increase in dMMN amplitude at 6-month follow-up visit also had better cognitive functioning, as compared to baseline values [90]. Conversely, the other group did not present an improvement in either dMMN amplitude or cognitive functions. Interestingly, one study using cognitive training in SCZ, showed that changes in dMMN (i.e., a decrease in latency), upon completion of just one hour of training, predicted improvement in verbal learning after a full cycle of treatment [172]. Furthermore, an additional study that focused on pre versus post cognitive training found that improvements in reasoning and problem-solving domains were associated with an increase in dMMN amplitude values [184].

However, other studies found no association of dMMN amplitude with memory [185], working memory [168,176], visual memory [185], semantic memory, decision making, cognitive control, learning capacities [185], executive functioning [168], attention [168,176,185], processing speed [193], facial emotion identification [183] or the capacity of making social inferences [195], as well as with general assessment of cognitive abilities [108,175,177] in SCZ, FEP and FES. A lack of association between dMMN and cognition was also reported by a study that did not find a predictive power of baseline dMMN amplitude for improvements in cognition upon completion of cognitive training therapy sessions [93].

##### Other Types of MMN Deviants

In addition to pitch and duration deviants, other studies employed deviant stimuli that were characterized, as compared to standard tones, by a different sound location (ear to which sounds were played to) or intensity. One study found that MMN amplitude elicited by deviant stimuli that were played from a different earphone as compared to standard ones was negatively correlated to speed of processing, verbal learning, visual learning and working memory in SCZ, while no significant correlations were observed in CHR subjects [177]. As to MMN amplitude elicited by stimuli deviant for intensity, negative correlations were reported with speed of processing, verbal learning and cognitive composite score (as assessed with the MCCB) [169,177].

#### 3.3.5. P200

The P200 component is a positive deflection with a typical peak latency of approximately 150–250 ms elicited by auditory, somatosensory and visual stimuli [241,242].

Most studies did not find a relationship between P200 and different cognitive functions, such as working memory [147,164], episodic memory [160], attention [147], executive functioning [147,196], speed of processing [196] and verbal and visual memory [147,196] in SCZ. However, a study reported a correlation between P200 amplitude and executive functioning in SCZ [162], and another one carried out in FEP subjects found a correlation with processing speed (Morales-Muñoz et al., 2017).

#### 3.3.6. N200

The N200 component is a negative-going component peaking between 200 and 350 ms after stimulus onset, which is often elicited in paradigms focused on visual attention and language processing [243].

Some studies did not find significant associations between N200 and cognitive domains as assessed through the MCCB [67,198,199,202], while other studies found that blunted N200 peaks were correlated to worse performance in memory [157,200], visual working memory [159] and attention [201] tasks in SCZ and FES. Furthermore, one study found that a less prominent N200 response was associated with better visuospatial attention in SCZ patients [156].

#### 3.3.7. P300

P300 is a positive peak that can be observed 300 ms after the presentation of a deviant or rare stimulus [244,245]. This ERP has been considered as an index of cognitive processing and attention shifts to changes in the environment and consistent deficits have been detected in its amplitude and latency in different stages of schizophrenia-spectrum disorders [56,246,247,248,249].

Most studies using different paradigms (visual, visuospatial memory and phonological tasks) reported a positive correlation of P300 amplitude reduction with an impairment in different cognitive abilities, such as visual attention [156,203,250], working memory [204,206], self-referential memory [207], language processing [206] in SCZ and with executive functioning in both SCZ and at-risk subjects [205]. Interestingly, a study that focused on the predictive value of ERPs in the efficacy of the combination of two cognitive remediation programs showed that higher baseline P300 amplitude values were associated with greater improvements in attention, memory and speed of processing [93]. However, two studies found no significant association of P300 amplitude with memory [164] or attention in social scenarios [158].

Several studies investigated P300 during auditory tasks and distinguished two different P300 values that present a distinct scalp topography and peak latency, suggesting different neural generators and association to different functions. In particular, the P3a component (230–350 ms after the stimulus onset), localized mainly in frontal cerebral regions, is elicited by presenting rare non-target stimuli and can be observed even under passive conditions and is related to novelty detection and attentional shifts [244]; the P3b component (275–600 ms after the stimulus onset), localized in temporo-parietal areas, is elicited by rare target stimuli when subjects are asked to perform a stimuli-related task, which reflects working memory updating and deficits in this component have been associated with difficulties in maintaining goal-directed behaviour [244,251].

##### P3a

Discrepant findings have been reported on associations between P3a amplitude and cognitive functions in SCZ, FEP and FES subjects. In particular, most studies reported a positive correlation between P3a amplitude and different cognitive domains, such as attention [180,192,197,208], verbal learning [115,180], working memory [96,115,180], non-verbal memory [96], executive functioning, abstraction and flexibility capacities [96] and social cognition [96,183,210]. Interestingly, one study testing the effects of cognitive training showed that the changes in P3a features (an amplitude increase and a latency decrease) upon completion of just one hour of training were significantly associated with improvements in verbal learning abilities after a full treatment cycle in SCZ subjects [172]. In addition, one study using low-resolution electromagnetic tomography analysis (LORETA) showed that the increase in P3a activity in the left superior temporal gyrus was associated with improvement in verbal learning after a six-month olanzapine treatment in SCZ subjects [209]. However, in SCZ, FES and FEP a lack of correlations between P3a amplitude and several cognitive domains were also reported [105,176,183,186,190,221].

##### P3b

Discrepancies are also found in the literature relevant to correlation between P3b and cognitive functions in SCZ, FES and high-risk subjects. Indeed, most studies found a positive correlation between P3b amplitude and several cognitive domains [91,157,162,163,197,200,201,204,208,211,212,214,215,216,217,218,219,220,221], while some studies found no significant correlation [161,210,213,220].

#### 3.3.8. N400

N400 is a negative deflection associated with language, faces, memory and visual processing [252]. Abnormalities in this ERP are present both during chronic [253] and early or prodromal stages of the illness [224]. Discrepant findings have been reported on correlations between N400 amplitude and cognitive functions. Indeed, some authors reported significant negative correlations of N400 amplitude with language comprehension [222], and the MCCB cognitive composite score [224] in SCZ, as well as with verbal learning and memory [223] in CHR. Other studies reported no significant correlations between N400 and episodic memory [160], executive functioning [196], speed of processing [196], verbal and visual memory [196] in SCZ.

#### 3.3.9. ERN and Pe

Error-related negativity (ERN) and error positivity (Pe) are two ERPs that have been associated with error detection, learning and conflict monitoring [254]. Error-related negativity (ERN) is a negative deflection ERP, occurring approximately 50–100 ms after an erroneous response and might be directly linked to the awareness of the error made [255]. The Pe reflects conscious error processing and is visualised as a positive-going component occurring from 200 to 500 ms following the erroneous response [256].

Studies involving subjects with SCZ [205,227] or those with genetic risk of developing schizophrenia [205] revealed that blunted ERN was associated with impaired executive [205,227], attention [227] and general cognitive functions [227]. However, also a lack of association between ERN amplitude and attention or cognitive control has been reported in SCZ [225]. As regards to Pe, in SCZ [205,227], CHR [205], or in subjects with a history of psychosis [226] blunted Pe was associated with impaired executive functioning [205,227], attention [227] and self-appraisal of task performance [226].

### 3.4. Sleep EEG Activity

Studies on electrophysiological activity during sleep in schizophrenia suggested that abnormalities during non-REM sleep represent indices of disturbances in cognitive functioning [257]. Physiologically, during non-REM sleep stages the frequency of EEG activity slows down. In addition, throughout non-REM stage 2 of sleep recurring phasic electrical events, such as sleep spindles and K-complexes have been reported [258,259]. Results of sleep studies are summarized in Table 4.

Sleep spindles, traditionally defined as waveforms between 12 and 14 Hz lasting up to 3 s in duration, are cortical signatures of the patterned thalamocortical and/or hippocampal-cortical network activity that supports the role of NREM sleep in overnight memory consolidation [269,270]. A reduction in the density of sleep spindles in SCZ was correlated to worse performance in tasks assessing memory skills after sleep [264,268], due to the role of sleep spindles in memory consolidation. Furthermore, another study highlighted that SCZ, as compared to healthy controls, presented abnormalities in the slow wave synchronization and density of sleep spindles [261]. Both indices predicted memory consolidation in healthy controls, but not in SCZ. Associations between sleep spindle characteristics and cognitive functions other than memory consolidation have also been observed [262,266]; however, a lack of associations has also been reported [260,263].

Furthermore, in a sample of subjects with SCZ, a study focusing on K-complexes (large electrical sharp waves in the EEG recordings) showed that a low number of K-complexes was associated with poor executive functioning and problem-solving skills [267]. A long duration of slow wave sleep was related to better problem solving [267], and a study focusing on nonlinear EEG complexity during sleep in FES reported that a low nonlinear complexity was associated with poor memory and deficits in executive functions [265].

## 4. Discussion

The examination of the alterations of EEG indices in subjects with schizophrenia-spectrum disorders may advance our understanding of the neural mechanisms underpinning cognitive impairments. However, discrepancies in the findings are numerous, possibly due to the heterogeneity in EEG parameters and cognitive measures employed across different studies, thus hindering the use of these measures in routine clinical practice.

### 4.1. Frequency Bands Activity

Studies that focused on frequency band analysis in subjects with schizophrenia suggest that aberrant cortical neural activity and failures in the synchronization of cerebral activity might underlie the impairments in different cognitive functions, such as memory, attention and cognitive control [82,271].

The studies included in the present review addressing delta activity at rest recorded not very robust findings (association with emotion recognition but not with attention, working memory, speed of processing, verbal and visual learning, reasoning or problem solving) [85,86], while those focussing on task-related delta activity, more consistently reported an association between reduced activity in this frequency band and impairments in attention and speed of processing [88,89,91].

Within the slow activity range, the band most robustly associated with cognitive deficits was theta, probably due to its role in the coordination of neural activity of the default-mode network, which is highly involved in automated processing of information [89,272], effortful cognitive processing and efficient allocation of attentive resources [98,102,107]. Furthermore, relationships between alterations in connectivity and measures of verbal memory and speed of processing suggest a possible role of disturbed communication across cortical areas and aberrant temporal synchronization of neuronal oscillators in the genesis of cognitive dysfunctions in schizophrenia [92,94,95,101,102].

Studies on resting state and evoked alpha power produced heterogeneous results [66,111,113,122], and it is therefore difficult to draw conclusions. Most of the studies, however, used standard alpha frequency instead of individual alpha peak frequency (IAPF) which may cause heterogeneity in the results of studies investigating the association of alpha frequency and cognitive functions. In fact, IAPF has been shown to be much more reliable and reproducible across sessions and cognitive tasks than standard alpha frequency measurements [113,273,274]. Studies investigating IAPF have reported a reduction in subjects with schizophrenia and some studies have hypothesized that dysfunctional timing of alpha waves could be at the core of defective cognitive processing [111,113,275]. Thus, the vast inter-subject’s variability in the standard frequency alpha range in both physiological and pathological conditions places some restrains in its use as assessment tools of cognitive impairments. Therefore, the hypothesis that defects in the coordination of alpha activity across brain networks could be responsible for inappropriate allocation of neuronal resources according to the cognitive load demand deserves further investigation using the IAPF and the most accurate method of assessment of this biomarker [273].

Finally, the high-frequency bands abnormalities in gamma activity have consistently been related to the impairment in various cognitive domains [82,98,116,117,119,120,121,122]. Deficits in synchronization of neural activity within the gamma frequency band might lead to cognitive dysfunctions by hindering efficient higher-order cognitive processes [82,98,116,117,119,120,121,122].

Overall, the results of the studies addressing frequency bands activity seem to suggest that deficits in the elicitation of balanced and synchronous oscillatory activity are often present in schizophrenia, and lead to difficulties in preserving both accurate and fast performance in the context of automated and effortful cognitive processing.

However, although frequency band analysis seems to offer manifold possibilities in evaluating cognitive dysfunctions, the main challenge in translating these measures into clinical tools remains the establishment of cut-off values that would flag the presence of cognitive impairments and the translation of results obtained at group level into application at the individual level. In the authors opinion, further studies, employing standardized paradigms and frequency ranges, using both power spectrum analysis and connectivity measures together with the implementation of machine learning approaches, might help to overcome the limitations of current findings.

### 4.2. ERPs

Investigations on correlations between cognitive functions and ERPs related to early sensory processing, such as P50, N100 and P100 led to discrepant results. However, most studies showed an association between these ERPs and several cognitive functions, such as attention, memory, learning, problem solving and executive functions [144,145,146,149,150,153,154,155,159,163,165,201]. It has been hypothesized that dysfunctions in auditory and visual sensory processing, as flagged by blunted amplitude of these EPRs, could derive from abnormalities in frontal and temporal regions and might contribute to impairments in higher-order cognitive functions [144,145,146,159]. Conversely, some studies found no association between P50, N100, P100 with cognitive skills [146,147,148,151,155,156,157,158,160,161,162,164].

A large body of research has been devoted to the relationship between cognitive deficits and abnormalities in MMN. Indeed, reductions in MMN amplitude, probably driven by abnormalities in frontal and temporal lobes [191], have consistently been linked to cognitive deficits and psychosocial functioning impairment in subjects with schizophrenia [179,276], independently from the clinical state [277]. Although the number of MMN studies included in the current review is high and results are more consistent as compared to other ERPs studies, drawing conclusions remains difficult. This might be due to the heterogeneity of sample sizes, of electrophysiological paradigms and of the assessment instruments used across different studies.

According to data reviewed in the present contribution, MMN recordings in schizophrenia seem to relate to deficits in encoding sensory information and storing information, as highlighted by the association between lower amplitude values and worse performance on memory tests [166]. Furthermore, since MMN is also a measure of salience of unexpected stimuli, the relationship between MMN measures and attention is probably due to a dysfunction in efficient filtering of relevant environmental stimuli in schizophrenia. Thus, according to current reviewed literature, both dMMN and pMMN could be considered as possible markers in the evaluation of memory and attentional deficits in schizophrenia. Interestingly, some studies on the efficacy of cognitive remediation therapies (CRTs) in subjects with schizophrenia suggested the potential role of MMN indices in monitoring and/or predicting the efficacy of these intervention in ameliorating cognitive dysfunctions [167,172,184].

The available literature on N200 and P200 is very heterogeneous, probably due to the use of different paradigms (e.g., reward-based, semantic, visual or acoustic tasks).

The P300 component, which is associated with the detection of novel stimuli, updating of working memory, inhibitory control, and selective attention processes [243], may be highly sensitive to deficits in cognition. As such, P300 has been one of the most studied ERP components in this context [278]. Associations between amplitude reductions in P300 [156,203] or its two subtypes, P3a [180,192,208] and P3b [201,208,211,215,216,218,219,220], with deficits in attention is one of the most consistent EEG finding in schizophrenia [156,163,192,201,203,208,215,218], first-episode psychosis [180,197,211] and clinical high-risk [216,219] subjects. Associations of P300 amplitude reduction with verbal learning and memory deficits have also been reported [96,115,157,162,180,200,206,217,218,220] and suggest that impairments in identifying and responding to stimuli that are either salient or novel might also influence higher-order steps of cognitive processing relevant to goal directed behaviour. Furthermore, studies involving CRTs have showed that P300 values could be used to predict patients’ responses to these interventions [93,172]. It was hypothesized that participants that presented larger reductions in P300 amplitude might benefit more from CRT, thanks to neuroplastic changes restoring cognitive processing.

Finally, more studies are required for other ERPs, such as N400, ERN and Pe to obtain a clearer insight on their associations with cognitive deficits.

In conclusion, ERPs studies provide an interesting perspective on the neuronal correlates of cognitive impairments due to their high temporal resolution. However, in order to obtain a clearer view on the results, we also need to consider that several factors might have influenced or biased the correlations reported. Few studies, in fact, have considered the possible confounding effects of illness duration, antipsychotic doses and severity of psychopathological aspects on the outcomes [63,79]. Furthermore, physiological processes such as ageing have also often been associated with a progressing deterioration of P300 and MMN elicitation [279,280].

Therefore, conducting studies on early stages of the illness, which are less biased by the effects of illness duration and medication usage, and the synergistic use of multiple ERPs might help to disentangle the complex biological mechanisms underlying cognitive deficits in schizophrenia.

### 4.3. Sleep EEG Activity

In the last two decades, studies focusing on sleep abnormalities measured through EEG recordings have suggested that sleep-related measures may improve our insight into the neurophysiological alterations leading to cognitive dysfunctions, and, especially, to memory impairments [281].

Most of the EEG sleep studies focused on the examination of sleep spindle density and characteristics of K-complexes that might be related to the disruption in memory consolidation and executive functions. Disturbances in sleep organization have mainly been attributed to disruption in the neural signalling of the thalamocortical network [261,263], which could lead to deficits in memory integration, information processing and consolidation of novel information [282,283]. However, so far, few studies have focused on associations between EEG sleep disturbances and cognitive dysfunctions in schizophrenia and results are not consistent [260,261,262,263,264,265,266,267,268]. Therefore, more studies are required to broaden the evidence currently available.

### 4.4. The Limits and Obstacles of EEG-Based Measures in Clinical Settings and Possible Strategies to Overcome Them

The results presented in the current review highlight how electrophysiological recordings might contribute to the evaluation of cognitive impairments in subjects with schizophrenia-spectrum disorders. However, limitations related to the use of this technique should be addressed.

Important limitations are the paucity of clinically relevant studies, the heterogeneity of employed methods and the frequent discrepancies in reported results. As a matter of fact, cognitive domains were evaluated using different scales or tasks; studies often reported the association with a total cognitive score and not with individual cognitive domains; a variety of EEG measurements/parameters is investigated: for instance a study focuses on alpha power, while another on alpha coherence; different experimental paradigms are used to record the same EEG index [165]; the samples included in different studies varied in terms of size, age range and illness-related variables, such as severity of negative and positive symptoms, or dosage of antipsychotic drugs [100,109,145,151]. This might be also due to the presence of flexible inclusion criteria in the current systematic review, which aimed at providing a broad and inclusive assessment of the available literature on the topic, with no exclusion criteria for duration of illness, type of EEG indices or preprocessing methodology. In addition, most studies reported data on one single EEG index or, if multiple EEG indices were recorded, statistical analyses were performed separately for each of them. In addition, few studies that investigated the potential of EEG indices in predicting the efficacy of rehabilitation interventions showed promising results. However, the paucity of data and the heterogeneity of methods used across studies, do not allow conclusions.

Therefore, we suggest some possible improvements and approaches for future studies to overcome these limitations.

Standardized assessment scales, such as the MCCB, which is considered the state-of-the-art instrument for cognitive evaluation in schizophrenia, should be used as the primary tool to evaluate the impairment in different cognitive domains.

Large research networks are needed to collect data by applying a common protocol in large patient cohorts, possibly with a longitudinal study design to investigate patterns of associations between changes in EEG parameters and cognitive performance over time. In addition, combining recordings of multiple EEG indices, rather than relying on a single electrophysiological measure, would definitely provide a more effective strategy. Machine learning methods have been increasingly adopted in psychiatry to optimize the use of neurobiological indices combined with other factors, such as clinical, behavioural, genetic and environmental data translation [284,285,286,287]. Some studies used the machine learning approach, also incorporating EEG measures to build classifiers able to discriminate patients from healthy controls or to predict disease trajectories [284,288,289,290,291,292], but very few EEG machine learning studies were conducted to investigate associations between cognitive impairments and EEG-based measures [90,99], and support the hypothesis that an objective and precise characterization of patient’s cognitive phenotypes.

**Figure 2:**
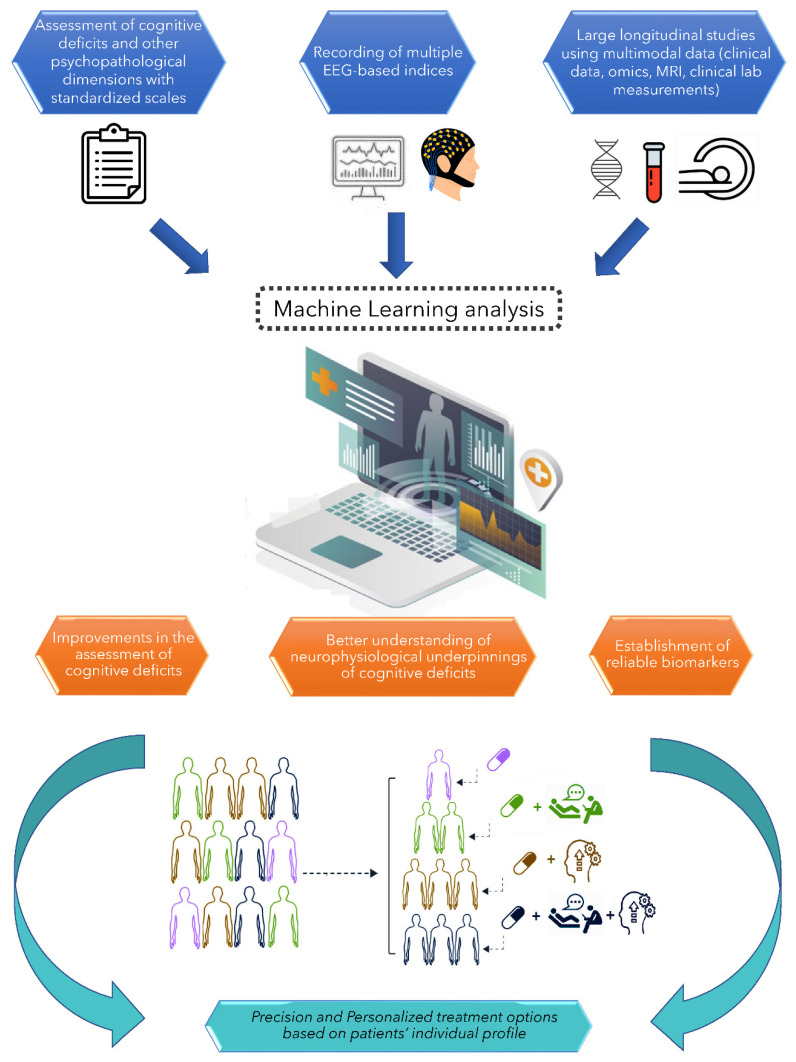
Future directions for the implementation of EEG-based measures in clinical settings. Large studies based on in depth characterization of the studied population, standardized methods, and adopting state of the art tools and indices, together with the implementation of machine learning approaches, might contribute to increase the use of EEG-based measures in clinical settings.

EEG-based measures have a potential for clinical translation; however, limitations, as discussed above, need to be carefully addressed. Large cross-sectional and longitudinal studies based on in depth clinical characterization of the studied population, standardized methods, and adopting state of the art tools and indices, together with the implementation of machine learning approaches, might contribute to increase the use of EEG-based measures in clinical settings (Figure 2).

## Figures and Tables

**Table 1:** Systematic search strategy.

Database	Search Syntax	Number of Retrieved Documents	Date of Search
PubMed	(EEG OR electroencephalography OR qEEG OR “quantitative EEG” OR “EEG microstate” OR “dipole source localization” OR sLORETA OR LORETA OR eLORETA OR ERP OR “event-related potential” OR “spectral analysis” OR “frequency domain analysis” OR “spectral band” OR “neural oscillations” OR “spectral power” OR “event-related” OR “evoked potential” OR “evoked-response”) AND (psychosis OR schizophrenia OR schizoaffective OR “first-episode psychosis” OR FEP OR “Ultra-High Risk” OR UHR OR Clinical High Risk OR CHR) AND (neurocognit* OR cognit* OR memory OR “verbal learning” OR “verbal memory” OR “visual learning” OR “visual memory” OR “visual-spatial learning” OR “visual-spatial memory” OR “working memory” OR attention OR vigilance OR “processing speed” OR “speed of processing” OR reasoning OR “problem solving” OR “Verbal executive function” OR “Cognitive flexibility” OR “Executive function*” OR Insight OR “Cognitive Perseveration” OR “Decision-making” OR Planning OR “Executive control” OR metacognit* OR Perseveration OR “error awareness” OR “error control” OR “error monitoring”)	1733	2 November 2021
Scopus	(EEG OR electroencephalography OR qEEG OR “quantitative EEG” OR “EEG microstate” OR “dipole source localization” OR sLORETA OR LORETA OR eLORETA OR ERP OR “event-related potential” OR “spectral analysis” OR “frequency domain analysis” OR “spectral band” OR “neural oscillations” OR “spectral power” OR “event-related” OR “evoked potential” OR “evoked-response”) AND (psychosis OR schizophrenia OR schizoaffective OR “first-episode psychosis” OR FEP OR “Ultra-High Risk” OR UHR OR Clinical High Risk OR CHR) AND (neurocognit* OR cognit* OR memory OR “verbal learning” OR “verbal memory” OR “visual learning” OR “visual memory” OR “visual-spatial learning” OR “visual-spatial memory” OR “working memory” OR attention OR vigilance OR “processing speed” OR “speed of processing” OR reasoning OR “problem solving” OR “Verbal executive function” OR “Cognitive flexibility” OR “Executive function*” OR Insight OR “Cognitive Perseveration” OR “Decision-making” OR Planning OR “Executive control” OR metacognit* OR Perseveration OR “error awareness” OR “error control” OR “error monitoring”)	460	2 November 2021
PsychINFO	(EEG OR electroencephalography OR qEEG OR “quantitative EEG” OR “EEG microstate” OR “dipole source localization” OR sLORETA OR LORETA OR eLORETA OR ERP OR “event-related potential” OR “spectral analysis” OR “frequency domain analysis” OR “spectral band” OR “neural oscillations” OR “spectral power” OR “event-related” OR “evoked potential” OR “evoked-response”) AND (psychosis OR schizophrenia OR schizoaffective OR “first-episode psychosis” OR FEP OR “Ultra-High Risk” OR UHR OR Clinical High Risk OR CHR) AND (neurocognit* OR cognit* OR memory OR “verbal learning” OR “verbal memory” OR “visual learning” OR “visual memory” OR “visual-spatial learning” OR “visual-spatial memory” OR “working memory” OR attention OR vigilance OR “processing speed” OR “speed of processing” OR reasoning OR “problem solving” OR “Verbal executive function” OR “Cognitive flexibility” OR “Executive function*” OR Insight OR “Cognitive Perseveration” OR “Decision-making” OR Planning OR “Executive control” OR metacognit* OR Perseveration OR “error awareness” OR “error control” OR "error monitoring”)	1357	2 November 2021

**Table 2:** Frequency bands activity studies.

Study	Cognitive Domains	EEG Indices	Sample Size	Correlations between EEG Indices and Cognitive Functions in Patients and High-Risk Subjects
**Delta activity**				
Dias et al., 2020 [84]	Attention/Vigilance, Working Memory, Speed of Processing, Verbal Learning, Visual Learning, Reasoning and Problem Solving (MCCB)	**Delta amplitude** *Task-related*	SCZ = 24 HCs = 25 Mean age: SCZ = 37 y; HC = 36 y	SCZ < HCs No significant correlation between evoked delta amplitude and cognitive domains
Gica et al., 2019 [85]	Emotion Recognition, Attention (CANTAB Reaction Time); Visual Memory (CANTAB Paired Associate Learning); Sustained Attention (Rapid Visual Information Processing); Planning (CANTAB One Touch Stockings of Cambridge); Flexible thinking (CANTAB Intra-Extra Dimensional Set Shift); Executive functions (CANTAB)	**Delta power** *Resting state*	SCZ = 24 (DSM-V) Mean age: 36 y	Negative correlation between delta power and emotion recognition
Koshiyama D. et al., 2021 [86]	Verbal Memory (CVLT); Working Memory (LNS)	**Delta power and PDI** *Resting state*	SCZ = 148 HCs = 143 Mean age: SCZ = 46 y; HCs = 40 y	**Delta power** SCZ > HCs No significant association between delta power and cognitive domains **Delta PDI** SCZ = HCs No significant association between delta PDI and cognitive domains
Martínez A. et al., 2019 [87]	Face-emotion recognition (FER) and Social Perception (behavioural task)	**Delta power** *Task-related*	SCZs = 19 HCs = 17 (SCID) Mean age: SCZ = 37 y; HCs = 34 y	SCZ < HCs Positive correlation between evoked delta power and social perception
Martínez et al., 2018 [88]	Attention/Vigilance, Working Memory, Speed of Processing, Verbal Learning, Visual Learning, Reasoning and Problem Solving Neurocognitive Composite Domains Score (MCCB)	**Delta power** *Task-related*	SCZ = 63 AP = 32 HCs = 44 (DSM-V; SIPS) Mean age: N.A.	SCZ and AP < HCs Positive correlation between evoked delta power with the cognitive composite score, visual learning, attention/vigilance and speed of processing
Prieto M et al., 2021 [89]	Working Memory, Immediate and Delayed Verbal Learning, Verbal Fluency, Speed of Processing and Psychomotor Speed (SCIP-S); Attention (D2 Test of Attention)	**Delta power** *Task-related*	SCZs = 22 HCs = 23 (ICD-10) Mean age: SCZ = 37 y; HCs = 39 y	SCZ = HCs Negative correlation between evoked delta power and working memory
Qu et al., 2020 [90]	Attention/Vigilance, Working Memory, Speed of Processing, Verbal Learning, Visual Learning, Reasoning and Problem Solving, Social Cognition (MCCB)	**Delta power** *Task-related*	FEP = 20 HCs = 33 (SCID; DSM-IV) Mean age: FEP = 22 y; HCs = 22 y	No correlation was found between delta event-related power and cognitive functions
Wu G. et al., 2021 [91]	Attention/Vigilance, Working Memory, Speed of Processing, Verbal Learning, Visual Learning, Reasoning and Problem Solving (MCCB)	**Delta power** **Delta ITC** *Task-related* *(auditory oddball task)*	CHR = 104 (SIPS) Mean age: 18 y	**Evoked Delta power** No correlations between evoked delta power and cognitive domains **Delta ITC** Positive correlation between Delta ITC and speed of processing
**Theta activity**				
Andreou et al., 2015 [92]	Memory (WMS); Attention (WAIS); Visuomotor Sequencing (TMT); Letter Fluency (RWT)	**Theta connectivity** *Resting state*	HR = 28 FES = 19 HCs = 23 (MINI, SPI, SIPS) Mean age: HR = 23 y; FES = 24 y; HCs = 25 y	FES > HCs Negative correlation between theta connectivity (within the bilateral orbitofrontal, medial frontal areas, posterior midline regions, sensorimotor areas and the temporoparietal junction) and verbal memory
Best et al., 2020 [93]	Neurocognitive composite score (MCCB)	**Theta power** *Task-related*	SCZ = 70 (SCID; DSM-IV) Mean Age: 37 y	Higher theta power at baseline was associated with greater improvement in neurocognitive composite score after completion of cognitive training sessions
Cea-Cañas et al., 2020 [94]	Working Memory, Speed of Processing, Executive Function, Verbal Memory, Motor Speed, Verbal Fluency, Speed of Processing (BACS)	**Theta Connectivity strength** *Task-related*	SCZ = 35 HCs = 51 (DSM-V) Mean age: SCZ = 36 y; HCs = 38 y	SCZ > HCs Negative correlation between theta connectivity strength and cognitive skills
Dias et al., 2020 [84]	Attention/Vigilance, Working Memory, Speed of Processing, Verbal Learning, Visual Learning, Reasoning and Problem Solving (MCCB)	**Theta** **amplitude** *Task-related*	SCZ = 24 HC = 25 Mean age: SCZ = 37 y; HC = 36 y	SCZ < HCs Positive correlation between theta evoked amplitude and attention and speed of processing
Gica et al., 2019 [85]	Emotion Recognition (CANTAB ERT); Attention (CANTAB Reaction Time); Visual Memory (CANTAB Paired Associate Learning); Sustained Attention (Rapid Visual Information Processing); Planning (CANTAB One Touch Stockings of Cambridge); Flexible thinking (CANTAB Intra-Extra Dimensional Set Shift); Executive functions (CANTAB Intra-Extra Dimensional Set Shift, One Touch Stockings of Cambridge and Spatial Working Memory)	**Theta power** *Resting state*	SCZ = 24 (DSM-V) Mean age: 36 y	Negative correlation between theta power and emotion recognition
Gomez-Pilar et al., 2018 [95]	Working memory, Speed of Processing, Executive Function, Verbal Memory, Motor Speed, Verbal Fluency (BACS)	**Theta Connectivity Modulation** *Task-related*	SCZ = 35 HCs = 51 (DSM-V) Mean age: SCZ = 33 y; HCs = 29 y	SCZ < HCs Positive correlation between theta connectivity modulation and speed of processing, verbal fluency and verbal memory
Hochberger et al., 2019 [96]	Executive Functions, Working Memory, Episodic Memory, Complex Cognitive Processing and Social Cognition (PENN CNB)	**Theta power** **Theta standard phase-locking** *Task-related*	SCZ = 706 HCs = 605 (DSM-IV, SCID-II) Mean age: SCZ = 46 y; HCs = 39 y	**Evoked theta activity** SCZ < HCs Positive correlation between theta evoked activity and social cognition **Theta standard phase-locking** SCZ < HCs Positive correlation between theta standard phase-locking and social cognition
Hochberger et al., 2020 [97]	Verbal Learning and cognitive composite score (MCCB)	**Theta power and phase-locking** *Task-related*	SCZ with Treatment as usual = 22 SCZ with Cognitive Training = 24 (SCID-DSM-IV) Mean age: 35 y	**Theta power** Higher baseline values were associated with greater improvements in global cognitive score after completing cognitive training sessions. Changes in theta power after only one hour of cognitive training were associated with greater improvements in verbal learning upon completion of a full treatment intervention **Theta phase-locking** Higher baseline values were associated with greater improvements in global cognitive score after completion of a full cognitive training intervention
Hoy et Al., 2021 [98]	Working Memory (Behavioural task)	**Theta power** *Task-related*	SCZs = 30 HCs = 27 (MINI) Mean age: SCZs = 46 y; HCs = 40 y	SCZ < HCs Positive correlation between task-related theta oscillations and working memory
Johannesen et al., 2016 [99]	Working Memory (SWMT and MCCB)	**Theta power** *Task-related*	SCZ = 40 HCs = 12 (DSM-IV) Mean Age: HCs = 43 y; SCZ = 46 y	Negative correlation between evoked theta power and working memory
Kirihara et al., 2012 [100]	Verbal Memory (CVLT); Executive Functions (WCST); Working Memory (LNS)	**Theta amplitude** *Task-related*	SCZ = 234 HCs = 188 (DSM-IV; SCID) Mean age: SCZ = 44 y; HC = 44 y	SCZ > HC Negative correlation between theta amplitude and verbal memory
Koshiyama D. et al., 2021 [86]	Verbal Memory (CVLT); Working Memory (LNS)	**Theta power and PDI** *Resting state*	SCZ = 148 HCs = 143 Mean age: SCZ = 46 y; HCs = 40 y	**Theta power** SCZ > HCs Negative correlation between theta power and verbal learning **Theta PDI** SCZ < HCs No significant association between theta PDI and cognitive domains
Krukow et al., 2018 [101]	Speed of processing (Naming Speed Test and Symbol Coding Test)	**Theta phase lag index** *Resting state*	FES = 32 HCs = 35 (DSM-V; SCID) Mean age: SCZ = 21 y; HC = 21 y	SCZ > HCs Negative correlation between of theta phase lag index of central regions and speed of processing
Krukow et al., 2020 [102]	Cognitive Initiation (Design fluency test)	**Theta band Synchronization strength** *Resting state*	FES = 34 HCs = 30 (DSM-V; SCID) Mean Age: FES = 21 y; HCs = 22 y	FES > HCs Negative correlation between theta hyper-connectivity/synchrony and cognitive initiation failure
Lee et al., 2020 [103]	Executive Functions (TMT); Verbal Memory (CVLT)	**Theta phase-gamma amplitude coupling** *Resting state*	FEP = 59 HCs = 50 (DSM-IV; SCID) Mean age: FEP = 23 y; HCs = 23 y	FEP > HCs Positive correlation between theta phase-gamma amplitude coupling in the left posterior cingulate cortex and executive functions and verbal memory
Liu et al., 2020 [104]	Working Memory (Visual Task)	**Theta power** *Task-related*	SCZ = 43 HCs = 57 (DSM-IV; SCID) Mean age: SCZ = 24 y; HCs = 24 y	SCZ< HCs Positive correlation between evoked theta power and working memory
Martínez A. et al., 2019 [87]	Face-emotion recognition (FER) and Social Perception (behavioural task)	**Theta power** *Task-related*	SCZs = 19 HCs = 17 (SCID) Mean age: SCZ = 37 y; HCs = 34 y	SCZ < HCs No correlation between evoked theta power and emotion recognition
Martínez et al., 2018 [88]	Attention/Vigilance, Working Memory, Speed of Processing, Verbal Learning, Visual Learning, Reasoning and Problem Solving, Neurocognitive Composite Domains Score (MCCB)	**Theta power** *Task-related*	SCZ = 63 AP = 32 HCs = 44 (DSM-V; SIPS) Mean age: NA	SCZ = AP = HCs Positive correlation between evoked theta power with visual learning and speed of processing.
Prieto M et al., 2021 [89]	Working Memory, Immediate and Delayed Verbal Learning, Verbal Fluency, Speed of Processing and Psychomotor Speed (SCIP-S); Attention (D2 Test of Attention)	**Theta power** *Task-related*	SCZs = 22 HCs = 23 (ICD-10) Mean age: SCZ = 37 y; HCs = 39 y	SCZ = HCs Positive correlation between evoked theta power, speed of processing and working memory.
Qu et al., 2020 [90]	Attention/Vigilance, Working Memory, Speed of Processing, Verbal Learning, Visual Learning, Reasoning and Problem Solving, Social Cognition (MCCB)	**Theta power** *Task-related*	FEP = 20 HCs = 33 (SCID-DSM-IV) Mean age: FEP = 22 y; HCs = 22 y	No correlation was found between event-related theta activity and cognitive performance
Solís-Vivanco R et al., 2021 [105]	Attention/Vigilance, Speed of Processing, and Working Memory and Cognitive total score (MCCB)	**Theta PLF and connectivity** *Task-related*	FEP = 15 HCs = 13 (DSM-IV) Mean age: FEP = 26 y HC = 23 y	**Theta PLF** FEP < HCs No significant correlations between theta PLF and cognitive domains **Theta connectivity** FEP = HCs No significant correlations between theta connectivity and cognitive domains
Grove et al., 2021 [106]	Attention (behavioural task); Working Memory, Speed of Processing, Executive Function, Verbal Memory, Motor Speed, Verbal Fluency; Speed of Processing (BACS)	**Theta power** *Task-related*	SCZ = 29 HCs = 44 (DSM-IV-TR) Mean age: SCZ = 42 y; HCs = 42 y	SCZ < HCs No significant correlation between evoked theta activity and total cognitive score
Wichniak et al., 2015 [107]	Attention/Vigilance, Working Memory, Speed of Processing, Verbal Learning, Visual Learning, Reasoning and Problem Solving (MCCB)	**Theta absolute power** *Resting state*	SCZ = 39 (ICD-10) Mean age: 28 y	Negative correlations between theta absolute power and verbal learning, visuospatial memory and executive functions
Xiong et al., 2019 [108]	Attention/Vigilance, Working Memory, Speed of Processing, Verbal Learning, Visual Learning, Reasoning and Problem Solving (MCCB)	**Theta amplitude** *Task-related*	SCZ = 40 FES = 40 HC = 40 (DSM-IV; SCID) Mean Age: SCZ = 29 y; FES = 26 y; HCs = 26 y	SCZ and FES < HCs (SCZ = FES) No significant correlations between evoked theta amplitude and cognitive domains
**Alpha activity**				
Adler et al., 2002 [109]	Attention/Vigilance (CPT); Working Memory (WAIS); Visual Memory (TCF); Verbal Memory (AVLT)	**Alpha Power** *Resting state*	SCZ = 17 (DSM-IV) Mean age: 30 y	Positive correlation between alfa power and working, visual and verbal memory
Billeke et al., 2015 [110]	Speed of Processing (Animal Naming and Symbol-Coding from the WAIS-III, and TMT-A), Attention/Vigilance (CPT-IP), Working Memory (LNS and Spatial Span from WMS-III), Visual Learning (Free Recall of RCFT And WMS-III), Planning and Reasoning (Copy of ROCF and Tol), and Social Cognition (Face Emotion Recognition Test)	**Alpha power** *Task-related*	SCZ = 20 HCs = 25 (DSM-IV, SCID-II) Mean age: SCZ = 28 y; HCs = 28 y	SCZ < HCs No significant correlations between evoked alpha activity and cognitive domains considered
Castelluccio et al., 2020 [111]	Neurocognitive Composite Score (MCCB)	**Alpha peak frequency** *Resting state*	SCZ = 37 (DSM-IV, SCID-II) Mean age: 46 y	At baseline, no significant associations between individual alpha peak frequency and assessment of cognitive measures assessed at baseline time point. However, A positive correlation between baseline Individual alpha peak frequency and improvements in cognition after completion of cognitive remediation treatment was detected
Gica et al., 2019 [85]	Emotion Recognition (CANTAB ERT); Attention (CANTAB Reaction Time); Visual Memory (CANTAB Paired Associate Learning); Sustained Attention (Rapid Visual Information Processing); Planning (CANTAB One Touch Stockings of Cambridge), Flexible thinking (CANTAB Intra-Extra Dimensional Set Shift); Executive functions (CANTAB Intra-Extra Dimensional Set Shift, One Touch Stockings of Cambridge and Spatial Working Memory).	**Alpha power** *Resting state*	SCZ = 24 (DSM-V) Mean age: 36 y	Positive correlation between alpha power and emotion recognition
Hoffman et al., 1991 [112]	Attention/Vigilance (CPT)	**Spatial EEG** Alpha coherence *Task-related*	SCZ = 13 HCs = 9 (DSM-III) Mean age: SCZ = 34 y; HCs = 34 y	SCZ < HCs Positive correlation between alpha coherence and vigilance
Johannesen et al., 2016 [99]	Working Memory (SWMT and MCCB)	**Alpha power** *Task-related*	SCZ = 40 HC = 12 (DSM-IV) Mean Age: HC = 43 y SCZ = 46 y	Positive correlation between evoked alpha power and working memory
Koshiyama D. et al., 2021 [86]	Verbal Memory (CVLT); Working Memory (LNS)	**Alpha power and** PDI *Resting state*	SCZ = 148 HCs = 143 Mean age: SCZ = 46 y; HCs = 40 y	**Alpha power** SCZ > HCs No significant association between alpha power and cognitive domains **Alpha PDI** SCZ > HCs No significant association between alpha PDI and cognitive domains
Krukow et al., 2018 [101]	Naming Speed Test and Symbol Coding Test (Speed of processing)	**Alpha phase lag index** *Resting state*	FES = 32 HCs = 35 (DSM-V; SCID) Mean age: SCZ = 21 y; HC = 21 y	FES < HCs Positive correlation between phase lag index of alpha and speed of processing
Liu et al., 2020 [104]	Working Memory (Visual Task)	**Alpha power** *Task-related*	SCZ = 43 HCs = 57 (DSM-IV; SCID) Mean age: SCZ = 24 y; HCs = 24 y	SCZ < HCs No significant correlation between evoked alpha power and working memory
Martínez A. et al., 2019 [87]	Face-emotion recognition (FER) and Social Perception (behavioural task)	**Alpha ERD amplitude** *Task-related*	SCZs = 19 HCs = 17 (SCID) Mean age: SCZ = 37 y; HCs = 34 y	SCZ < HCs No correlation between alpha ERD amplitude and emotion recognition or motion-sensitivity
Prieto M et al., 2021 [89]	Working Memory, Immediate and Delayed Verbal Learning, Verbal Fluency, Speed of Processing and Psychomotor Speed (SCIP-S); Attention (D2 Test of Attention)	**Alpha amplitude** *Task-related*	SCZs = 22 HCs = 23 (ICD-10) Mean age: SCZ = 37 y; HCs = 39 y	SCZ = HCs Positive correlation between alpha amplitude and speed of processing
Qu et al., 2020 [90]	Attention/Vigilance, Working Memory, Speed of Processing, Verbal Learning, Visual Learning, Reasoning and Problem Solving, Social Cognition (MCCB)	**Alpha power** *Task-related*	FEP = 20 HCs = 33 (SCID-DSM-IV) Mean age: FEP = 22 y; HCs = 22 y	No correlation between alpha evoked activity and cognitive domains
Ramsay I.S. et Al., 2021 [113]	Speed of Processing, Perceptual Reasoning, Working Memory and Verbal Reasoning (WAIS-III); Attention-Vigilance (CPT)	**Individual alpha peak frequency (IAPF** ***)*** *Resting state*	SCZs = 104 HCs = 101 (DSM–IV; SCID) Mean age: SCZ = 42 y; HCs = 45 y	SCZ < HCs Positive correlation between IAPF and speed of processing, perceptual reasoning, attention-vigilance, working memory and verbal reasoning
Vignapiano et al., 2019 [66]	Attention/Vigilance, Working Memory, Speed of Processing, Verbal Learning, Visual Learning, Reasoning and Problem Solving Neurocognitive Composite Domains Score (MCCB)	**Alpha Power** *Resting state*	SCZ = 145 HC = 69 (DSM-IV SCID) Mean Age: SCZ = 37 y; HC = 36 y	SCZ < HCs No correlation between alpha power amplitude and speed of processing, attention/vigilance, working memory, verbal learning, visual learning, reasoning, problem solving and neurocognitive composite score
**Beta activity**				
Adler et al., 2002 [109]	Attention/Vigilance (CPT); Working Memory (WAIS); Visual Memory (TCF); Verbal Memory (AVLT)	**Beta power** *Resting state*	SCZ = 17 (DSM-IV) Mean age: 30 y	Positive correlation between beta power and vigilance, working, visual and verbal memory
Briley Paul M. et al., 2021 [114]	Working memory (n-back task-related)	**Beta bursts** *Task-related*	SCZs = 32 HCs = 30 Mean age of the whole sample: 34 y	SCZ < HCs Positive correlation between beta bursts and working memory
Gica et al., 2019 [85]	Emotion Recognition (CANTAB ERT); Attention (CANTAB Reaction Time); Visual Memory (CANTAB Paired Associate Learning); Sustained Attention (Rapid Visual Information Processing); Planning (CANTAB One Touch Stockings of Cambridge); Flexible thinking (CANTAB Intra-Extra Dimensional Set Shift); Executive functions (CANTAB Intra-Extra Dimensional Set Shift, One Touch Stockings of Cambridge and Spatial Working Memory)	**Beta power** *Resting state*	SCZ = 24 (DSM-V) Mean age: 36 y	Positive correlation between beta power and emotion recognition
Johannesen et al., 2016 [99]	Working Memory (SWMT and MCCB)	**Beta power** *Task-related*	SCZ = 40 y HCs = 12 y (DSM-IV) Mean Age: HCs = 43 y; SCZ = 46 y	Positive correlation between beta power and working memory
Koshiyama D. et al., 2021 [86]	Verbal Memory (CVLT); Working Memory (LNS)	**Beta power and** PDI *Resting state*	SCZ = 148 HCs = 143 Mean age: SCZ = 46 y; HCs = 40 y	**Beta power** SCZ > HCs No significant association between beta power and cognitive domains **Beta PDI** SCZ = HCs No significant association between beta PDI and cognitive domains
**Gamma activity**				
Hoy et Al., 2021 [98]	Working Memory (behavioural task)	**Gamma power** *Task-related*	SCZs = 30 HCs = 27 (MINI) Mean age: SCZs = 46 y; HCs = 40 y	SCZ = HCs Negative correlation between evoked theta oscillations and working memory
Johannesen et al., 2016 [99]	Working Memory (SWMT and MCCB)	**Gamma power** *Task-related*	SCZ = 40 HC = 12 (DSM-IV) Mean age: HC = 43 y; SCZ = 46 y	Negative correlation between evoked gamma activity and working memory
Koshiyama D. et al., 2021 [86]	Verbal Memory (CVLT); Working Memory (LNS)	**Gamma power and** **PDI** *Resting state*	SCZ = 148 HCs = 143 Mean age: SCZ = 46 y; HCs = 40 y	**Gamma power** SCZ > HCs No significant association between gamma power and cognitive domains **Gamma PDI** SCZ = HCs No significant association between gamma PDI and cognitive domains
Koshiysma D et al., 2021 [115]	Verbal Memory (CVLT); Working Memory (LNS)	**Gamma-band-ASSR** *Task-related*	SCZs = 695 HCs = 503 (DSM-IV; SCID) Mean age: SCZ = 45 y; HC = 44 y	SCZ < HCs Positive correlation between gamma-band-ASSR and working memory
Lee et al., 2020 [103]	Executive Functions (TMT); Verbal Memory (CVLT)	**Theta phase-gamma amplitude coupling** *Resting state*	FEP = 59 HCs = 50 (DSM-IV; SCID) Mean age: FEP = 23 y; HCs = 23 y	FEP > HCs Positive correlation between theta phase-gamma amplitude coupling in the left posterior cingulate cortex and psychomotor, executive functions and verbal memory
Leicht et al., 2015 [116]	Verbal Memory and Learning (WMS, VLMT), Attention (WAIS); Working Memory (WAIS); Visuomotor Sequencing (TMT); Letter Fluency (LFT)	**Gamma power** *Task-related*	FES = 23 HCs = 22 (MINI) Mean age: FES = 26 y; HCs = 22 y	FES < HCs Positive correlation between evoked gamma activity and working memory
Light et al., 2006 [117]	Verbal memory (CVLT); Executive Functions (WCST); Working Memory (LNS)	**Gamma ITC** *Task-related*	SCZ = 100 HCs = 80 (DSM-IV; SCID) Mean age: SCZ = 46 y; HCs = 34 y	SCZ < HCs Positive correlation between gamma ITC and working memory. No correlation with verbal memory, concept formation and conceptual flexibility
Molina et al., 2020 [118]	Neurocognitive composite score (MCCB)	**Gamma power** *Task-related*	SCZ -Treatment as usual (TAU) = 21 SCZ -Cognitive Training = 21 (SCID-DSM-IV) Mean age: SCZ-TAU = 33 y; SCZ-Cognitive training = 36 y	Higher baseline values in evoked power were predicting greater improvements in neurocognitive composite score after competition of full cognitive training intervention
Ramyead et al., 2015 [119]	Nonverbal/abstract reasoning abilities (LPS)	**CSD Gamma activity** *Resting state*	ARMS = 63 HCs = 29 (BSIP) Mean age: ARMS = 26 y; HCs = 22 y	ARMS > HCs Positive correlation between CSD gamma activity and non-verbal reasoning
Sun et al., 2018 [120]	Attention, Working Memory, Speed of Processing, Verbal Learning, Visual Learning, Reasoning and Problem Solving (MCCB)	**Gamma PLF and ITPC** *Task-related*	SCZ = 24 HCs = 30 (DSM-IV) Mean age: SCZ = 33 y; HCs = 34 y	SCZ < HCs Positive correlation between gamma PLF and ITPC with reasoning and problem solving
Tanaka-Koshiyama et al., 2020 [121]	Single-word reading ability (WRAT); Auditory attention and working memory (LNS); Executive functioning (WCST); Verbal learning and memory performance (CVLT)	**Gamma power** *Task-related*	SCZ = 157 HCs = 145 (DSM-IV-TR) Mean age: SCZ = 46 y; HCs = 40 y	SCZ > HCs Negative correlation between evoked gamma activity with verbal learning and memory performance
Vohs et al., 2015 [122]	Metacognitive functions: understanding of one’s own mind; understanding of others’ minds; decentration; metacognitive mastery (MAS-A)	**Gamma power** *Resting state*	SCZ = 20 (DSM-IV; SCID) Mean age: 43 y	Negative correlation between gamma power and decentration capacity
Williams et al., 2009 [123]	Emotional Intelligence (BRIEF), Negativity Bias (Neo-FFI), Emotion Identification (Facial Emotion Perception Task)	**Gamma synchrony** *Task-related*	FES = 28 HCs = 28 (DSM-IV, SCID) Mean age: SCZ = 20 y; HCs = 20 y	FES > HCs Negative correlation between gamma and measures of social cognition (emotion identification, negativity bias and emotional intelligence)

The column “EEG Indices” reports the EEG index considered in that study, while in the last column, differences between patients and controls, as well as correlations with cognition for that measure are reported; in *italics* the EEG recording method used (task or resting). Attenuated psychotic syndrome (AP): auditory steady-state responses (ASSR); Rey’s Auditory Verbal Learning Test (AVLT); Brief Assessment in Cognition in Schizophrenia (BACS); Resource Inventory for Emotional intelligence Factors (BRIEF); Basel Screening Instrument for Psychosis (BSIP); Continuous Performance Test (CPT); Current source density (CSD); California Verbal Learning Test (CVLT); Diagnostic and Statistical Manual of Mental Disorder (DSM); Emotion Recognition Test (ERT); Event-related spectral perturbation (ERSP); subjects with first-episode psychosis (FEP); subjects with first-episode schizophrenia (FES); Global Assessment of Functioning scale (GAF); High-risk for psychosis (HR); Kullback–Leibler Modulation Index (KLMI); Individual alpha peak frequency (IAPF); Inter-trial phase coherence (ITC); Inter-trial phase coherence (ITPC); Letter fluency test (LFT); Low Resolution Brain Electromagnetic Tomography (LORETA); Letter-Number Sequencing test (LNS); Leistungsprüfsystem Scale (LPS); Metacognition Assessment Scale-Abbreviated (MAS-A); Mini International Neuropsychiatric Interview (MINI); Not available (NA); NEO Personality Inventory (NEO-FII); Phase Discontinuity Index (PDI); Phase Locking factor (PLF); Phase-locking value (PLV); Rey-Osterrieth Complex Figure test (ROCF); Regensburger Lexical Fluency Test (RWT); Schizophrenia Proneness Instrument (SPI); Sternberg working memory task (SWMT); Structured Clinical Interview for DSM (SCID); Structured Interview for Prodromal Syndromes (SIPS); Taylor Complex Figure test (TCF); Trail Making Test (TMT); Verbal Learning and Memory Test (VLMT); The Wechsler Adult Intelligence Scale (WAIS); Wisconsin Card Sorting Test (WCST); Wechsler Memory Scale (WMS); Years (Y); Young controls (YC).

**Table 3:** Event-related potential studies.

Study	Cognitive Domains	EEG Indices	Sample Size	Correlations between EEG Indices and Cognitive Functions in Patients and High-Risk Subjects
**P50**				
Cullum et al., 1993 [144]	Verbal Learning and memory (CVLT); Vigilance (WAIS)	**P50 Ratio**	SCZ = 14 HCs = 15 (DSM-III) Mean age: SCZ = 35 y; HCs = 29 y	SCZ > HCs Negative correlation between P50 ratio and vigilance
Hamilton et al., 2018 [145]	Attention/Vigilance, Working Memory, Speed of Processing, Verbal Learning, Visual Learning, Reasoning and Problem Solving (MCCB)	**P50 Ratio**	SCZ = 39 HCs = 45 (SCID, DSM-IV) Mean age: SCZ = 26 y; HCs = 30 y	SCZ > HCs Negative correlation between P50 ratio and working memory and speed of processing. No significant correlations with the other cognitive domains analysed
Hseich et al., 2004 [146]	Visual Memory and Visual Learning (WMS)	**P50 Ratio**	SCZ = 10 HCs = 10 (DSM-IV) Mean age: SCZ = 35 y; HCs = 33 y	SCZ > HCs Negative correlation between P50 ratio and visual learning
Şahin D. et al., 2021 [147]	Working memory, executive function, information processing speed, learning and attention (Cognitive Basis Assessment Test battery)	**P50 amplitude**	SCZ = 35 HCs = 35 (DSM-IV) Mean age: SCZ = 28 y; HCs = 29 y	SCZ = HCs No significant correlation between P50 amplitude and cognitive functions
Sánchez-Morla et al., 2013 [148]	Speed of Processing (TMT; WAIS-R, Category Verbal Fluency Test); Verbal Working Memory (WMS); Attention (CPT); Verbal Learning and Memory (CVLT); Visual Memory (RCFT); Executive Functions (WCST, Stroop Test, TMT and COWA)	**P50 Ratio** **P50 Difference**	SCZ = 38 HCs = 32 (SCID, DSM-IV) Mean age: SCZ = 44 y; HCs = 37 y	**P50 Ratio** SCZ > HCs No correlation between P50 ratio and any cognitive domains **P50 Difference** SCZ = HCs No correlation between P50 difference and any cognitive domains
Smith et al., 2010 [149]	Attention (TMT, CPT); Working Memory (WCST, WAIS); Verbal Memory (AVLT, WMS)	**P50 Ratio**	SCZ = 79 HCs = 73 (SCID; DSM-IV) Mean age: SCZ = 43 y; HCs = 41 y	SCZ > HCs Negative correlation between P50 ratio and attention and working memory performance. No correlation with long-delay memory
Toyomaki et al., 2015 [150]	Executive Functions (WCST); Verbal Fluency (WFT); Sustained Attention and Motor Speed (CPT); Visual-Motor Processing and Motor Speed (TMT); Verbal Learning and Immediate and Recent Memory (AVLT); Selective Attention (Stroop Test)	**P50 S1 Amplitude** **P50 S2 Amplitude** **P50 Ratio**	SCZ = 41 (SCID; DSM-IV) Mean age: 29 y	**P50 S1 Amplitude** No significant correlations between S1 amplitude and cognitive domains **P50 S2 Amplitude** Negative correlation between P50 S2 amplitude and the performance during the task **P50 Ratio** Negative correlation between P50 ratio and executive functions. No significant correlations with the other cognitive domains
Xia et al., 2020 [151]	Attention, Working Memory, Speed of Processing, Verbal Learning, Visual Learning, Reasoning and Problem Solving (MCCB)	**P50 S1 Amplitude** **P50 S2 Amplitude** **P50 Ratio**	SCZ = 183 HCs = 116 (SCID; DSM-IV) Mean age: SCZ = 46 y; HCs = 45 y	**P50 S1 amplitude** SCZ < HCs No correlation between the P50 S1 amplitude and any cognitive domains **P50 S2 amplitude** SCZ = HCs No correlation between the P50 S2 amplitude and any cognitive domains **P50 ratio** SCZ > HCs No correlation between the P50 ratio and any cognitive domains.
Xia et al., 2020 [152]	Immediate Memory, Visuospatial/Constructional, Language, Attention, and Delayed Memory (RBANS)	**P50 S1 Amplitude** **P50 S2 Amplitude** **P50 Ratio**	SCZ = 38 HCs = 32 (SCID, DSM-IV) Mean age: SCZ = 31 y; HCs = 34 y	**P50 S1 amplitude** SCZ < HCs No correlation between the P50 S1 amplitude and any cognitive domains **P50 S2 amplitude** SCZ > HCs No correlation between the P50 S2 amplitude and any cognitive domains **P50 ratio** SC = HCs No correlation between the P50 ratio and any cognitive domains
**N100**				
Arjona-Valladares A. et al., 2021 [153]	Working Memory (Test Performance); Working Memory, Speed of Processing, Executive Function, Verbal Memory, Motor Speed, Verbal Fluency, Speed of Processing and Problem Solving (BACS); Executive Functions (WCST)	**N100 amplitude**	SCZ = 250 HCs = 35 (DSM-V) Mean age: SCZ = 38 y; HCs = 34 y	SCZ > HCs Negative correlation between N100 amplitude and cognitive performance during the task
Boutros et al., 2009 [154]	Executive Functions (WCST)	**N100 Ratio**	SCZ = 40 HCs = 46 (SCID; DSM-IV) Mean age: SCZ = 45 y; HCs = 39 y	SCZ > HCs Negative correlation between N100 and executive functions
Brodeur et al., 2016 [155]	Attention (RBANS)	**N100 amplitude**	SCZ = 16 HCs = 20 (SCID; DSM-IV) Mean age: SCZ = 44 y; HCs = 39 y	SCZ = HCs No significant correlation between N100 amplitude and attention
Bruder et al., 1998 [156]	Visuospatial Attention (dot enumeration task)	**N100 amplitude**	SCZ = 28 HCs = 28 (DSM-IV) Mean age: SCZ = 33 y; HCs = 36 y	SCZ > HCs No significant correlation between N100 amplitude and visuospatial attention
Bruder et al., 2001 [157]	Verbal, Logical and Visual Memory (WMS)	**N100 amplitude**	SCZ = 40 HCs = 14 (MINI) Mean age: SCZ = 33 y; HCs = 32 y	SCZ = HCs No significant correlation between N100 amplitude and memory
Catalano et al., 2021 [158]	Social Attention (Behavioural Task)	**N100 amplitude**	SCZ = 36 HCs = 20 (DSM-V: SCID) Mean age: SCZ = 48 y; HCs = 51 y	SCZ = HCs No significant correlation between N100 amplitude and the performance during the social attention task
Dias et al., 2011 [159]	Attention/Vigilance (CPT); Working Memory (Task)	**N100 amplitude**	SCZ = 15 HCs = 27 (DSM-IV; SCID) Mean age: SCZ = 33 y; HCs = 33 y	SCZ > HCs Negative correlation between N100 amplitude and the performance during the working memory task
Green et al., 2017 [160]	Episodic memory (Memory Task Performance)	**N100 amplitude**	SCZ = 24 HCs = 19 (MINI) Mean age: 37 y	SCZ = HCs No significant correlation between N100 amplitude and episodic memory
Hseich et al., 2004 [146]	Visual Memory and Visual Learning (WMS)	**N100 Ratio**	SCZ = 10 HCs = 10 (DSM-IV) Mean age: SCZ = 35 y; HCs = 33 y	SCZ = HCs No significant correlation between N100 ratio and visual learning
Kim et al., 2003 [161]	Visual Memory (RCFT); Verbal Fluency (COWA); Executive Functions (WCST and TMT); Verbal Memory and Learning (WAIS)	**N100 amplitude**	SCZ = 22 HCs = 21 (SCID-DSM-IV) Mean age: SCZ = 30 y; HCs = 28 y	SCZ = HCs No significant correlation between N100 amplitude and cognitive domains
Nagasawa et al., 1999 [162]	Visual and Verbal Memory and Learning (WMS); Executive Functions (TMT)	**N100 amplitude**	SCZ = 28 HCs = 30 DSM-III Mean age: SCZ = 24 y; HCs = 25 y	SCZ > HCs No significant correlation between N100 amplitude and cognitive domains
Şahin D. et al., 2021 [147]	Working memory, executive function, information processing speed, learning and attention (Cognitive Basis Assessment Test battery)	**N100 amplitude**	SCZ = 35 HCs = 35 (DSM-IV) Mean age: SCZ = 28 y; HCs = 29 y	SCZ > HCs No significant correlation between N100 amplitude and cognitive domains
Smith et al., 2010 [149]	Attention (TMT, CPT), Working Memory (WCST, WAIS), Verbal Memory (AVLT, WMS).	**N100 Ratio**	SCZ = 79 HCs = 73 (SCID; DSM-IV) Mean age: SCZ = 43 y; HCs = 41 y	SCZ > HCs Negative correlation between N100 ratio and attention and working memory. No significant correlation between N100 ratio and long-delay memory
Sumich et al., 2008 [163]	Verbal Memory (WMS-R and RAVLT), Executive Functions (WCST, Verbal fluency, Stroop Colour Word and TMT)	**N100 amplitude**	SCZ = 18 HCs = 18 (DSM-III) Mean age: SCZ = 31 y; HCs = 28 y	SCZ > HCs Negative correlation between N100 amplitude and verbal recall, immediate and delayed visual memory
Zhao et al., 2011 [164]	Different phases of working memory, including early visual processing and late memory-related processes of encoding, maintenance, and retrieval (SMST paradigm)	**N100 amplitude**	SCZ = 67 HCs = 46 Mean age: SCZ = 42 y; HCs = 39 y	SCZ > HCs No significant correlation between N100 amplitude and working memory performance
**P100**				
Brodeur et al., 2016 [155]	Attention (RBANS)	**P100 amplitude**	SCZ = 16 HCs = 20 (SCID; DSM-IV) Mean age: SCZ = 44 y; HCs = 39 y	SCZ < HCs Positive correlation between P100 amplitude and attention
Bruder et al., 1998 [156]	Visuospatial Attention (dot enumeration task)	**P100 amplitude**	SCZ = 28 HCs = 28 (DSM-IV) Mean age: SCZ = 33 y; HCs = 36 y	SCZ = HCs No significant correlation between P100 amplitude and visuospatial attention
Spironelli et al., 2019 [165]	Verbal processing (phonological task)	**P100 amplitude**	SCZ = 18 HCs = 30 (DSM-IV; SCID) Mean age: SCZ = 39 y; HCs = 53 y	SCZ > HCs Negative correlation between P100 amplitude and phonological processing
Zhao et al., 2011 [164]	Different phases of working memory, including early visual processing and late memory-related processes of encoding, maintenance, and retrieval (SMST paradigm)	**P100 amplitude**	SCZ = 67 HCs = 46 Mean age: SCZ = 42 y; HCs = 39 y	SCZ = HCs (in encoding phase of the task); SCZ < HCs (in retrieval phase of the task) No significant correlation between P100 amplitude and working memory performance
**pMMN**				
Baldeweg et al., 2015 [166]	Everyday memory (MMSE and RBMT); Working Memory (WAIS-R); Executive Control and Semantic Retrieval Verbal Fluency (FAS Score from COWA Test), Pre-Morbid Verbal Intelligence (NART)	**pMMN amplitude**	SCZ = 49 HCs = 49 (ICD-10) Mean age: SCZ = 38 y; HCs = 36 y	SCZ > HCs Negative correlations between pMMN amplitude and everyday memory and verbal fluency
Biagianti et al., 2017 [167]	Cognitive composite score (MCCB)	**pMMN amplitude**	SCZ = 56 HCs = 105 SCID (DSM-IV) Mean age = NA	SCZ > HCs No significant correlation between pMMN amplitude at baseline and cognitive score after completion of a full cycle of cognitive training treatment
Brockhaus-Dumke et al., 2005 [168]	Verbal Memory (AVLT); Verbal Executive Functions (Verbal fluency); Spatial Working Memory (DRT); Attention/Vigilance (CPT); Executive Functions (WCST); Verbal Intelligence (MWT)	**pMMN amplitude**	SCZ = 31 Prodromal subjects = 43 HCs = 33 (DSM-IV; SCID) Mean age: SCZ = 26 y; prodromal subjects = 25 y; HCs = 24 y	SCZ = Prodromal subjects = HCs No significant correlation between pMMN amplitude and cognitive domains
Carrión et al., 2015 [169]	Reading ability (GORT-4), CTOPP, and WRAT-3). Attention/Vigilance, Working Memory, Speed of Processing, Verbal Learning, Visual Learning, Reasoning and Problem Solving (MCCB)	**pMMN amplitude**	CHR = 17 HCs = 18 (SCID-DSM-IV) Mean age: SCZ = 39 y; HCs = 38 y	CHR > HCs Negative correlation between pMMN amplitude and speed of processing and verbal learning
Csukly et al., 2013 [170]	Emotion Recognition (FEEST)	**pMMN amplitude**	SCZ = 24 HCs = 24 (DSM-V) Mean age: SCZ = 34 y; HCs = 33 y	SCZ > HCs Negative correlation between pMMN amplitude and emotion recognition
Haigh et al., 2016 [171]	Cognition Composite Score (MCCB); Global Cognition (BACS)	**pMMN amplitude**	SCZ = 27 HCs = 27 (SCID-DSM-IV) Mean age: SCZ = 36 y; HCs = 32 y	SCZ > HCs Negative correlation between pMMN amplitude and working memory
Hochberger et al., 2019 [172]	Attention/Vigilance, Working Memory, Speed of Processing, Verbal Learning, Visual Learning, Reasoning and Problem Solving (MCCB)	**pMMN amplitude**	SCZ = 22 (SCID-DSM-IV) Mean age: SCZ = 36 y	No significant correlation between changes in pMMN values (baseline and follow-up evaluations) and changes in cognitive skills after a full treatment cycle of cognitive training
Kantrowitz et al., 2015 [173]	Auditory Emotion Recognition (emotional prosody task)	**pMMN amplitude**	SCZ = 43 HCs = 36 (DSM-IV) Mean age: NA	SCZ > HCs Negative correlation between pMMN amplitude and auditory emotion recognition
Kargel et al., 2014 [174]	Premorbid verbal intelligence (MWT-B); Verbal Fluency (WFT); Visual Speed of Processing and motor implementation of visual information (TMT-A); Cognitive Switching Or Flexibility (TMT-B); (WAIS); Speed of Processing (the Digit Symbol Test); Verbal Working Memory (the Digit Span Test); Auditory Verbal Memory (WLMPR (immediate and delayed retrieval)	**pMMN latency**	SCZ = 40 HCs = 16 (SCID; DSM-IV) Mean age: SCZ = 39 y; HCs = 38 y	SCZ > HCs Positive correlation between pMMN latency and verbal working memory
McCleery et al., 2019 [175]	Cognitive Composite Score (MCCB)	**pMMN amplitude**	SCZ = 43 HCs = 30 (DSM-IV; SCID) Mean age: SCZ = 49 y; HCs = 46 y	SCZ > HCs No significant correlation between pMMN amplitude and cognitive domains
Randau et al., 2019 [176]	Working memory (BACS), Attention (IED)	**pMMN amplitude**	FEP = 56 HCs = 64 (ICD-10) Mean age: SCZ = 25 y; HCs = 25 y	FEP = HCs No significant correlation between pMMN amplitude and working memory or attention
Sehatpour et al., 2021 [177]	Attention/Vigilance, Working Memory, Speed of Processing, Verbal Learning, Visual Learning, Reasoning and Problem Solving, Neurocognitive Composite Domains Score (MCCB)	**pMMN amplitude**	SCZ = 42 CHR = 33 HCs = 28 (DSM-V) Mean age: SCZ = 35 y; CHR = 22 y; HCs = 34 y	SCZ > HCs; CHR = HCs No significant correlation between pMMN amplitude and cognitive domains
Todd et al., 2014 [178]	Premorbid Intelligence (WTAR); the Vocabulary and Matrix Reasoning subtests of WASI; Working Memory (LNS task and DS subtests from WMS); Contextual Processing (CPT-AX).	**pMMN amplitude**	SCZ = 33 HCs = 30 (ICD-10) Mean age: SCZ = 44 y; HCs = 41 y	SCZ > HCs No significant correlation between pMMN amplitude and cognitive domains
Xiong et al., 2019 [108]	Attention/Vigilance, Working Memory, Speed of Processing, Verbal Learning, Visual Learning, Reasoning and Problem Solving (MCCB)	**pMMN amplitude**	SCZ = 40 FES = 40 HC = 40 (DSM-IV; SCID) Mean Age: SCZ = 29 y; FES = 26 y; HCs = 26 y	SCZ > HCs; FES = HCs Positive correlation between pMMN amplitude and composite MCCB score in FES but not SCZ
**dMMN**				
Baldeweg et al., 2004 [179]	Everyday Memory (MMSE And RBMT); Verbal Memory (WMS); Verbal Fluency (FAS Score from COWA Test); Pre-Morbid Verbal Intelligence (NART)	**dMMN amplitude**	SCZ = 42 HCs = 20 (ICD-10) Mean age: SCZ = 42 y; HCs = 39 y	SCZ > HCs No correlation between dMMN amplitude and cognitive domains
Baldeweg et al., 2015 [166]	Everyday memory (MMSE and RBMT); Working Memory (WAIS-R); Executive Control and Semantic Retrieval Verbal Fluency (FAS Score from COWA Test); Pre-Morbid Verbal Intelligence (NART)	**dMMN amplitude**	SCZ = 49 HCs = 49 (ICD-10) Mean age: SCZ = 38 y; HCs = 36 y	SCZ > HCs Negative correlation between dMMN amplitude and everyday memory and verbal fluency
Best et al., 2020 [93]	Neurocognitve composite score (MCCB)	**dMMN amplitude**	SCZ = 70 (SCID-DSM-IV) Mean age: 37 y	dMMN amplitude at baseline did not predict change in any of the cognitive or measures after completion of cognitive training sessions
Biagianti et al., 2017 [167]	Cognitive composite score (MCCB)	**dMMN amplitude**	SCZ = 56 HCs = 105 SCID (DSM-IV) Mean age: NA	SCZ > HCs Lower dMMN amplitude at baseline, predicted greater improvements of cognitive score after completion of a full cycle of cognitive training treatment.
Brockhaus-Dumke et al., 2005 [168]	Verbal Memory (AVLT); Verbal Executive Functions (Verbal fluency); Spatial Working Memory (DRT); Attention/Vigilance (CPT); Executive Functions (WCST); Verbal Intelligence (MWT)	**dMMN amplitude**	SCZ = 31 Prodromal subjects = 43 HCs = 33 (DSM-IV; SCID) Mean age: SCZ = 26 y; prodromal subjects = 25 y; HCs = 24 y	SCZ > prodromal subjects > HCs No significant correlation between dMMN amplitude and cognitive domains
Carrión et al., 2015 [169]	Reading ability (GORT-4, CTOPP, and WRAT-3), Attention/Vigilance, Working Memory, Speed of Processing, Verbal Learning, Visual Learning, Reasoning and Problem Solving (MCCB)	**dMMN amplitude**	CHR = 17 HCs = 18 (SCID-DSM-IV) Mean age: SCZ = 39 y; HCs = 38 y	SCZ > HCs Negative correlation between dMMN amplitude and speed of processing and verbal learning
Haigh et al., 2016 [171]	Cognitive composite score (MCCB); Global Cognition (BACS)	**dMMN amplitude**	SCZ = 27 HCs = 27 (SCID-DSM-IV) Mean age: SCZ = 36 y; HCs = 32 y	SCZ > HCs No significant correlation between dMMN amplitude and any cognitive domains
Hermens et al., 2010 [180]	Premorbid Intelligence (WTAR); Speed of Processing (TMT-A); Executive Functions (TMT-B); Attention (MC Subtest of the WMS); Verbal Learning and Memory (RAVLT)	**dMMN amplitude**	FEP = 17 HCs = 17 (DSM-IV) Mean age: FEP = 22 y; HCs = 23 y	FEP > HCs Negative correlation between dMMN amplitude and attention. Positive correlation between dMMN amplitude and speed of processing
Higgins et al., 2021 [181]	Attention/Vigilance, Working Memory, Speed of Processing, Verbal Learning, Visual Learning, Reasoning and Problem Solving, Neurocognitive Composite Score (MCCB); Social Cognition (TASIT-II and MSCEIT of MCCB)	**dMMN amplitude**	SCZ = 24 HCs = 42 (DSM-IV, SCID) Mean age: SCZ = 23 y; HCs = 24 y	SCZ > HCs Negative correlation between dMMN amplitude and social cognition at follow-up after 12 months
Higuchi et al., 2013 [182]	Verbal Memory, Working Memory, Motor Function, Verbal Fluency, Attention and Processing Speed, Executive Function (BACS-J)	**dMMN amplitude**	ARMS = 17 (converters = 13, non-converters = 4) FEP = 20 SCZ = 11 HCs = 20 (CAARMS; DSM-IV; SCID) Mean age: ARMS = 19 y; FEP = 27 y; SCZ = 28 y; HCs = 25 y	SCZ > HCs; ARMS = HCs Negative correlation between dMMN amplitude and verbal fluency
Hochberger et al., 2019 [96]	Executive Functions, Working Memory, Episodic Memory, Complex Cognitive Processing, Speed of Processing, and Social Cognition (PENN CNB)	**dMMN amplitude**	SCZ = 706 HCs = 605 (DSM-IV; SCID-II) Mean age: SCZ = 46 y; HCs = 39 y	SCZ > HCs Negative correlation between dMMN amplitude and executive functions (abstraction and flexibility), working memory, non-verbal memory and social cognition.
Hochberger et al., 2019 [172]	Attention/Vigilance, Working Memory, Speed of Processing, Verbal Learning, Visual Learning, Reasoning and Problem Solving, Neurocognitive Composite Score (MCCB)	**dMMN latency**	SCZ = 22 (SCID-DSM-IV) Mean age = 36 y	Changes in dMMN peak latency after one-hour training significantly predicted changes in verbal learning post full treatment
Jahshan et al., 2013 [183]	Emotional affective prosody, Facial Emotion Identification Task (behavioural task)	**dMMN amplitude**	SCZ = 36 HCs = 18 (DSM-IV, SCID-II) Mean age: SCZ = 48 y; HCs = 46 y	SCZ > HCs Negative correlation between dMMN amplitude and emotional affective prosody
Jahshan et al., 2019 [184]	Attention/Vigilance, Working Memory, Speed of Processing, Verbal Learning, Visual Learning, Reasoning and Problem Solving (MCCB)	**dMMN amplitude**	SCZ = 99 SCID (DSM-IV) Mean age: 51 y	Negative Correlation between baseline MMN and cognitive composite score. Negative Correlation between improvements in dMMN and improvements in Reasoning and Problem Solving domain after completion of cognitive training treatment
Kaser et al., 2013 [185]	Attention, Executive functions, Memory, Social and Emotion Cognition (CANTAB)	**dMMN amplitude**	SCZ = 20 HCs = 20 (MINI) Mean age: SCZ = 34 y; HCs = 32 y	SCZ > HCs No significant correlation between MMN amplitude and cognitive domains
Kaur et al., 2011 [186]	Speed of Processing (TMT); Attentional Switching (TMT); Attention (MC Subtest of the WMS); Verbal Learning and Memory (RAVLT).	**dMMN amplitude**	FEP = 18 HCs = 18 (SCID-DSM-IV) Mean age: SCZ = 22 y; HCs = 23 y	FEP > HCs Negative correlation between dMMN amplitude and attention, verbal learning and attentional switching
Kargel et al., 2014 [174]	Premorbid verbal intelligence (MWT-B); Verbal Fluency (WFT); Visual Speed of Processing and motor implementation of visual information (TMT A); Cognitive Switching or Flexibility (TMT B); (WAIS), Speed of Processing (the Digit Symbol Test); Verbal Working Memory (the Digit Span Test); Auditory Verbal Memory (WLMPR (immediate and delayed retrieval)	**dMMN amplitude dMMN latency**	SCZ = 40 HCs = 16 (SCID-DSM-IV) Mean age: SCZ = 39 y; HCs = 38 y	SCZ > HCs No correlation between dMMN amplitude and peak latency with cognitive domains
Kawakubo et al., 2006 [187]	Verbal Learning (RAVLT)	**dMMN amplitude**	SCZ = 14 (DSM-IV) Mean age: 28 y	Negative correlation between dMMN amplitude and verbal learning
Koshiysma D et al., 2021 [115]	Verbal Learning (CVLT); Working Memory (LNS)	**dMMN amplitude**	SCZs = 695 HCs = 503 (DSM-IV; SCID) Mean age: SCZ = 45 y HC = 44 y	SCZ > HCs Negative correlation between dMMN amplitude and verbal learning and working memory
Lee et al. 2014 [188]	Verbal Fluency (Verbal Fluency Test - Animal); Symbol Coding (Adapted from the BACS); Visual Attention (TMT); Executive Functions (TMT-B); Theory of Mind (Cartoon Test, False Beliefs, Physical Story and Tom Story)	**dMMN amplitude**	SCZ = 25 HCs = 29 (SCID-DSM-IV) Mean age: SCZ = 36 y; HCs = 30 y	SCZ > HCs Negative correlation between dMMN amplitude and visual attention
Lho et al., 2020 [189]	Speed of processing (TMT)	**dMMN amplitude**	FEP = 25 HCs = 22 (SCID; DSM-IV) Mean age: FEP = 26 y; HCs = 24 y	FEP > HCs An increase in dMMN amplitude over a 1-year period (more blunted amplitude) in FEP correlated to worsening in speed of processing
Light et al., 2015 [190]	Global Cognition (MMSE)	**dMMN amplitude**	SCZ = 877 HCs = 754 (SCID-DSM-IV) Mean age: SCZ = 42 y; HCs = 39 y	SCZ > HCs Negative correlation between dMMN amplitude and global cognitive score
McCleery et al., 2019 [175]	Cognitive composite score (MCCB)	**dMMN amplitude**	SCZ = 43 HCs = 30 (DSM-IV; SCID) Mean age: SCZ = 49 y; HCs = 46 y	SCZ > HCs No significant correlation between dMMN amplitude and cognitive composite score
Miyanishi et al., 2013 [191]	Verbal Memory, Working Memory, Motor Function, Verbal Fluency, Attention and Processing Speed, Executive Function (BACS-J)	**dMMN amplitude**	SCZ = 20 HCs = 20 (DSM-IV; SCID) Mean age: SCZ = 25 y; HCs = 27 y	SCZ > HCs Negative correlation between dMMN amplitude and working memory
Qu et al., 2020 [90]	Attention/Vigilance, Working Memory, Speed of Processing, Verbal Learning, Visual Learning, Reasoning and Problem Solving, Social Cognition (MCCB)	**dMMN amplitude**	FEP = 20 HCs = 33 (SCID-DSM-IV) Mean age: FEP = 22 y; HCs = 22 y	The machine learning model showed that one group of subjects that presented an increase in dMMN amplitude at 6-month follow visit had also better values on cognitive functions, as compared to baseline values. Conversely, the other group did not present an improvement in neither dMMN amplitude or cognitive functions
Randau et al., 2019 [176]	Working memory (BACS); Attention (IED)	**dMMN amplitude**	FEP = 56 HCs = 64 (ICD-10) Mean age: SCZ = 25 y; HCs = 25 y	SCZ > HCs No correlation between dMMN amplitude and peak latency with working memory and attention
Rissling et al., 2013 [192]	Attention/Vigilance (CPT-IP)	**dMMN amplitude**	SCZ = 20 HCs = 20 (SCID-DSM-IV) Mean age: SCZ = 50 y; HCs = 48 y	SCZ > HCs Negative correlation between dMMN mean amplitude and attention/vigilance
Rowland et al., 2016 [193]	Verbal Working Memory (digital span task); Speed of Processing (digit Symbol Coding subtest of the WAIS)	**dMMN amplitude**	SCZ = 45 HCs = 53 (SCID-DSM-IV) Mean age: SCZ = 38 y; HCs = 37 y	SCZ > HCs Positive correlation between dMMN amplitude and verbal working memory
Sehatpour et al., 2021 [177]	Attention/Vigilance, Working Memory, Speed of Processing, Verbal Learning, Visual Learning, Reasoning and Problem Solving, Neurocognitive Composite Score (MCCB)	**dMMN amplitude**	SCZ = 42 CHR = 33 HCs = 28 (DSM-V) Mean age: SCZ = 35 y; CHR = 22; HCs = 34 y	SCZ > HCs; CHR = HCs No significant correlation between dMMN amplitude and neurocognitive composite score in SCZ but not in CHR
Todd et al., 2014 [178]	Premorbid Intelligence (WTAR); the Vocabulary and Matrix Reasoning subtests of WASI; Working Memory (LNS task and DS subtests from WMS); Contextual Processing (CPT-AX)	**dMMN amplitude**	SCZ = 33 Matched HC = 30 (ICD-10) Mean age: SCZ = 44 y; HCs = 41 y	SCZ > HCs Negative correlation between dMMN amplitude and contextual processing
Toyomaki et al., 2008 [194]	Executive functions (WCST); Verbal Fluency (WFT); Attention and Motor Speed (CPT); Visual-Motor Processing and Speed of Processing (TMT); Response Inhibition And Selective Attention (Stroop Test); Error (TMT)	**dMMN amplitude** **dMMN latency**	SCZ = 23 (DSM-IV) Mean age: 31 y	**dMMN amplitude** Negative correlation between mean dMMN amplitude and executive functions, response inhibition and selective attention, visual-motor processing and speed of processing **dMMN latency** Negative correlation between dMMN latency and speed of processing
Wynn et al., 2010 [195]	Social Perception (PONS), Social inferences (TASIT)	**dMMN amplitude**	SCZ = 33 HCs = 42 (DSM-IV, SCID-II) Mean age: SCZ = 41 y; HCs = 33 y	SCZ > HCs Negative correlation between dMMN amplitude and social perception. No correlation between dMMN amplitude and the capacity of making social inferences
Xiong et al., 2019 [108]	Attention/Vigilance, Working Memory, Speed of Processing, Verbal Learning, Visual Learning, Reasoning and Problem Solving (MCCB)	**dMMN amplitude**	SCZ = 40 FES = 40 HC = 40 (DSM-IV; SCID) Mean Age SCZ = 29 y; FES = 26 y; HCs = 26 y	SCZ and FES > HCs No significant correlation between dMMN amplitude and cognitive domains in FES and SCZ
***Intensity and Location MMN deviants***				
Carrión et al., 2015 [169]	Reading ability (GORT-4), CTOPP, and WRAT-3). Attention/Vigilance, Working Memory, Speed of Processing, Verbal Learning, Visual Learning, Reasoning and Problem Solving (MCCB)	**Intensity MMN**	CHR = 17 HCs = 18 (SCID-DSM-IV) Mean age: SCZ = 39 y; HCs = 38 y	SCZ > HCs Negative correlations between MMN intensity and speed of processing and verbal learning
Sehatpour et al., 2021 [177]	Attention/Vigilance, Working Memory, Speed of Processing, Verbal Learning, Visual Learning, Reasoning and Problem Solving, Neurocognitive Composite Score (MCCB)	**Location deviant MMN** **Intensity deviant MMN**	SCZ = 42 CHR = 33 HCs = 28 (DSM-V) Mean age: SCZ = 35 y; CHR = 22 y; HCs = 34 y	**Location deviant MMN** SCZ and CHR > HCs Negative correlation between location deviant MMN and neurocognitive composite score, speed of processing, verbal learning, visual learning and working memory in SCZ, but not in CHR **Intensity deviant MMN** SCZ and CHR = HCs Negative correlation between intensity MMN and neurocognitive composite score in SCZ but not in CHR
**P200**				
Favre et al., 2020 [196]	Executive function, Speed of Processing, Verbal and Visual Memory (Cogstate/DRM Paradigm)	**P200 amplitude**	SCZ = 25 HCs = 24 (ICD-10) Mean age: SCZ = 23 y; HCs = 22 y	SCZ < HCs No significant correlation between P200 amplitude and cognitive domains
Green et al., 2017 [160]	Episodic memory (Memory Task Performance)	**P200 amplitude**	SCZ = 24 HCs = 19 (MINI) Mean age: 37 y	SCZ = HCs No significant correlation between P200 amplitude and episodic memory
Morales-Muñoz et al., 2017 [197]	Attention/Vigilance, Working Memory, Speed of Processing, Verbal Learning, Visual Learning, Reasoning and Problem Solving (MCCB)	**P200 amplitude**	FEP = 38 HCs = 38 (DSM-IV; SCID) Mean age: FEP = 27 y; HCs = 30 y	FEP < HCs Negative correlation between P200 amplitude and speed of processing
Nagasawa et al., 1999 [162]	Visual and Verbal Memory and Learning (WMS); Executive Functions (TMT)	**P200 amplitude**	SCZ = 28 HCs = 30 (DSM-III) Mean age: SCZ = 24 y; HC = 25 y	SCZ = HCs Negative correlation between P200 amplitude and executive functions
Şahin D. et al., 2021 [147]	Working memory, executive function, information processing speed, learning and attention (Cognitive Basis Assessment Test battery)	**P200 amplitude**	SCZ = 35 HCs = 35 (DSM-IV) Mean age: SCZ = 28 y; HCs = 29 y	SCZ < HCs No significant correlation between P200 amplitude and cognition
Zhao et al., 2011 [164]	Different phases of working memory, including early visual processing and late memory-related processes of encoding, maintenance, and retrieval (SMST paradigm)	**P200 amplitude**	SCZ = 67 HCs = 46 Mean age: SCZ = 42 y; HCs = 39 y	SCZ < HCs No significant correlation between P200 amplitude and working memory performance
**N200**				
Bruder et al., 1998 [156]	Visuospatial Attention (dot enumeration task)	**N200 amplitude**	SCZ = 28 HCs = 28 (DSM-IV) Mean age: SCZ = 33 y; HCs = 36 y	SCZ < HCs Positive correlations between N200 amplitude and visuospatial attention
Bruder et al., 2001 [157]	Verbal, Logical and Visual Memory (WMS)	**N200 amplitude**	SCZ = 40 HCs = 14 (MINI) Mean age: SCZ = 33 y; HC = 32 y	SCZ > HCs Negative correlation between N200 amplitude and logical memory
Coffman et al., 2016 [198]	Attention, Working Memory, Speed of Processing, Verbal Learning, Visual Learning, Reasoning, Problem Solving and Social Cognition (MCCB)	**N200 amplitude**	SCZ = 24 HCs = 22 (SCID DSM-IV) Mean age: SCZ = 36 y; HCs = 32 y	SCZ > HCs No significant correlation between N200 amplitude cognitive domains.
Coffman et al., 2018 [199]	Attention, working memory, speed of processing, verbal learning, visual learning, reasoning and problem solving (MCCB)	**N200 amplitude**	FES = 20 HCs = 24 SCID (DSM-IV) Mean age: FES = 23 y; HCs = 25 y	FES = HCs No significant correlation between N200 amplitude and cognitive domains
Dias et al., 2011 [159]	Attention/Vigilance (CPT); Working Memory (Task)	**N200 amplitude**	SCZ = 15 HCs = 27 (DSM-IV; SCID) Mean age: SCZ = 33 y; HCs = 33 y	SCZ > HCs Negative correlation between N200 amplitude and the performance on working memory task
Kayser et al., 1999 [200]	Verbal Memory (behavioural task)	**N200 amplitude**	SCZ = 24 HCs = 19 (DSM-IV) Mean age: SCZ = 33 y; HCs = 32 y	SCZ > HCs Negative correlation between N200 amplitude and verbal memory
Klein S. D. et al., 2020 [201]	Attention/Vigilance (CPT)	**N200 amplitude**	SCZ = 48 HCs = 68 (DSM-IV) Mean age: SCZ = 46 y; HCs = 45 y	SCZ < HCs Negative correlation between N200 amplitude and attention/vigilance
Sklar Alfredo et al., 2020 [202]	Visual Attention (task performance); MCCB Total Score	**N200 amplitude**	FES = 32 HCs = 32 (SCID-DSM-IV) Mean age: FES = 22 y; HC = 22 y	FES > HCs No significant correlation between N200 amplitude and visual attention or MCCB total score
Vignapiano et al., 2018 [67]	Attention, Working Memory, Speed of Processing, Verbal Learning, Visual Learning, Reasoning and Problem Solving (MCCB)	**N200 amplitude**	SCZ = 22 HCs = 34 (MINI) Mean age: SCZ = 33 y; HCs = 32 y	SCZ = HCs No significant correlation between N200 amplitude and cognitive domains
**P300**				
Best et al., 2020 [93]	Neurocognitive composite score (MCCB)	**P300 amplitude**	SCZ = 70 (SCID-DSM-IV) Mean age: 37 y	Positive Correlation between P300 amplitude measured at baseline and improvements in neurocognitive composite score at follow-up, after completion of cognitive training sessions
Bruder et al., 1998 [156]	Visuospatial Attention (dot enumeration task)	**P300 amplitude**	SCZ = 28 HCs = 28 (DSM-IV) Mean age: SCZ = 33 y; HCs = 36 y	SCZ < HCs Positive correlations between P300 amplitude and visuospatial attention
Catalano et al., 2021 [158]	Social Attention (Behavioural Task)	**P300 amplitude**	SCZ = 36 HCs = 20 (DSM-V: SCID) Mean age: SCZ = 48 y; HCs = 51 y	SCZ = HCs No significant correlation between P300 amplitude and the performance during the social attention task
Clementz et al., 2008 [203]	Attention (Visual task)	**P300 amplitude**	SCZ = 17 HCs = 17 SCID (DSM-IV) Mean age: SCZ = 43 y; HCs = 41 y	SCZ < HCs Positive correlation between P300 amplitude and the performance on the visual attention task
Ditcher et al., 2006 [204]	Information, Picture Completion, and Digit Span subtests of the WAIS-R; Executive Functions (TMT-A and B, TOL-version A, CPT); Visuospatial Working Memory (WCST)	**P300 amplitude**	SCZ = 13 HCs = 12 (SCID-DSM-IV) Mean age: SCZ = 28 y; HCs = 30 y	SCZ < HCs Positive correlation between P300 amplitude and working memory
Francisco et al., 2020 [205]	Executive Functions (D-KEFS)	**P300 amplitude**	22 q11.2 DS = 27 SCZ = 15 HCs(matched to 22 q11.2 DS) = 27 HCs (matched to SCZ) = 15 Mean age: 22 q11.2 DS and matched HCs = 22 y; SCZ and matched HCs = 43 y	SCZ and 22 q11.2 DS < HCs Positive correlation between P300 amplitude and executive functions in both sample groups.
Wang et al., 2017 [206]	Phonological Processing (Tone Judgement Task), Working Memory (Digit Span Task)	**P300 amplitude**	SCZ = 47 HCs = 48 (DSM-IV) Mean age: SCZ = 28 y; HCs = 25 y	SCZ < HCs Positive correlation between P300 amplitude and phonological processing and working memory
Zhao et al., 2011 [164]	Different phases of working memory, including early visual processing and late memory-related processes of encoding, maintenance, and retrieval (SMST paradigm)	**P300 amplitude**	SCZ = 67 HCs = 46 Mean age: SCZ = 42 y; HCs = 39 y	SCZ > HCs No significant correlation between P300 amplitude and working memory performance
Zhao et al., 2014 [207]	Self-Referential Memory (Memory Task)	**P300 amplitude**	SCZ = 21 HCs = 22 (SCID-DSM-IV) Mean age: SCZ = 25 y; HCs = 25 y	SCZ = HCs Negative correlation between P300 amplitude and self-referential memory
**P3a**				
Andreasen et al., 2016 [208]	Attention/Vigilance (CPT), Executive Functions (D-KEFS)	**P3a amplitude**	SCZ = 31 HCs = 47 (DSM-IV) Mean age: SCZ = 27 y; HCs = 25 y	SCZ = HCs Positive correlation between P3a amplitude and attention
Hermens et al., 2010 [180]	Premorbid Intelligence (WTAR); Speed of Processing (TMT-A); Executive Functions (TMT-B); Attention (MC Subtest of the WMS); Verbal Learning and Memory (RAVLT): immediate recall (sum of trials 1–5; RAVLT A1 to A5), interference (distracter trial; RAVLT B1), post-interference recall (trial 6; RAVLT A6) and 20 min delayed recall (trial 7; RAVLT A7)	**P3a amplitude**	FEP = 17 HCs = 17 (DSM-IV) Mean age: FEP = 22 y; HCs = 23 y	FEP < HCs Positive correlations between P3a amplitude and attention and verbal learning and memory
Higuchi et al., 2008 [209]	Japanese Verbal Learning Test (JVLT); and Digit Span from the Wechsler Adult Intelligence Scale Revised (WAIS)	**P3a** (topographical activity through LORETA analysis)	SCZ = 16 HCs = 16 (SCID-DSM-IV) Mean age: SCZ = 31 y; HCs = 31 y	SCZ > HCs Correlations between the increase in LORETA values of left superior temporal gyrus and verbal learning memory after treatment with olanzapine
Hochberger et al., 2019 [96]	Executive Functions, Working Memory, Episodic Memory, Complex Cognitive Processing, Speed of Processing, and Social Cognition (PENN CNB)	**P3a amplitude**	SCZ = 706 HCs = 605 (DSM-IV; SCID-II) Mean age: SCZ = 46 y; HCs = 39 y	SCZ < HCs Positive correlation between P3a amplitude and executive functions (abstraction and flexibility), working memory, non-verbal memory and social cognition
Hochberger et al., 2019 [172]	Attention, working memory, speed of processing, verbal learning, visual learning, reasoning and problem solving (MCCB)	**P3a amplitude and peak latency**	SCZ = 22 (SCID-DSM-IV) Mean age = 36 y	Changes in P3a features (an amplitude increase and a latency decrease) upon completion of just one hour of cognitive training were significantly associated with improvements in verbal learning abilities after a full treatment cycle
Jahshan et al., 2013 [183]	Emotional affective prosody, Facial Emotion Identification Task (behavioural task)	**P3a amplitude**	SCZ = 36 HCs = 18 (DSM-IV; SCID-II) Mean age: SCZ = 48 y; HCs = 46 y	SCZ < HCs Positive correlation between P3a amplitude and emotional affective prosody
Johnston et al., 2005 [210]	Emotion recognition (facial emotion recognition task)	**P3a amplitude**	SCZ = 10 HCs = 15 (ICD-10) Mean age: SCZ = 31 y; HCs = 30 y	SCZ = HCs Positive correlation was between P3a amplitude and emotion recognition
Kaur et al., 2011 [186]	Speed of Processing (TMT); Attentional Switching (TMT); Attention (MC Subtest of the WMS); Verbal Learning and Memory (RAVLT)	**P3a amplitude**	FEP = 18 HCs = 18 (SCID-DSM-IV) Mean age: SCZ = 22 y; HCs = 23 y	FEP < HCs No significant correlation between P3a amplitude and cognitive domains
Koshiysma D et al., 2021 [115]	Verbal Learning (CVLT); Working Memory (LNS)	**P3a amplitude**	SCZs = 695 HCs = 503 (DSM-IV; SCID) Mean age: SCZ = 45 y HC = 44 y	SCZ < HCs No significant correlation between P3a amplitude and verbal learning and working memory
Kruiper et al., 2019 [211]	Working Memory, Attention, Executive Functions (CANTAB)	**P3a amplitude**	FES = 73 HCs = 93 (ICD-10; DSM-IV) Mean age: FES = 25 y; HCs = 26 y	FES < HCs No significant correlation between P3a amplitude and cognitive domains
Light et al., 2015 [190]	Global Cognitive score (MMSE)	**P3a amplitude**	SCZ = 877 HCs = 754 (SCID-DSM-IV) Mean age: SCZ = 42 y; HCs = 39 y	SCZ > HCs No correlation between P3a amplitude and the global cognitive score
Morales-Muñoz et al., 2017 [197]	Attention/Vigilance, Working Memory, Speed of Processing, Verbal Learning, Visual Learning, Reasoning and Problem Solving (MCCB)	**P3a amplitude**	FEP = 38 HCs = 38 (DSM-IV; SCID) Mean age: FEP = 27 y; HCs = 30 y	FEP < HCs Positive correlation between P3a amplitude and attention/vigilance
Randau et al., 2019 [176]	Working memory (BACS), Attention (IED)	**P3a amplitude and peak latencies**	FEP = 56 HC = 64 (ICD-10) Mean age: SCZ = 25 y; HCs = 25 y	SCZ < HCs No correlation between P3a amplitude and latency with working memory and attention
Rissling et al., 2013 [192]	Attention/Vigilance (CPT-IP)	**P3a amplitude**	SCZ = 20 HCs = 20 (SCID-DSM-IV) Mean age: SCZ = 50 y; HCs = 48 y	SCZ < HCs Positive correlation between pP3a amplitude and attention/vigilance
Solís-Vivanco et al., 2021 [105]	Attention/Vigilance, Speed of Processing, and Working Memory and Cognitive composite score (MCCB)	**P3a amplitude**	FEP = 15 HCs = 13 (DSM-IV) Mean age: FEP = 26 y; HCs = 23 y	FEP < HCs No significant correlations between P3a and MCCB scores
**P3b**				
Andreasen et al., 2016 [208]	Attention/Vigilance (CPT); Executive Functions (D-KEFS)	**P3b amplitude**	SCZ = 31 HCs = 47 (DSM-IV) Mean age: SCZ = 27 y; HCs = 25 y	SCZ < HCs Positive correlation between P3b amplitude and executive functioning and attention.
Bruder et al., 2001 [157]	Verbal, Logical and Visual Memory (WMS)	**P3b amplitude**	SCZ = 40 HCs = 14 MINI Mean age: SCZ = 33 y; HCs = 32 y	SCZ = HCs No significant correlation between P3b amplitude verbal memory
Chang et al., 2014 [212]	Visual, Verbal and General Memory (WMS)	**P3b amplitude**	SCZ = 14 HCs = 14 (SCID-DSM-IV) Mean age: SCZ = 25 y; HCs = 26 y	SCZ = HCs Positive correlation between P3a amplitude and visual and general memory
Ditcher et al., 2006 [204]	Information, Picture Completion, and Digit Span subtests of the WAIS-R; Executive Functions (TMT-A and B, TOL-version A, CPT); Visuospatial Working Memory (WCST)	**P3b amplitude**	SCZ = 13 HCs = 12 (SCID-DSM-IV) Mean age: SCZ = 28 y; HCs = 30 y	SCZ < HCs Positive correlation between P3b amplitude and working memory and executive functions
Ertekin et al., 2017 [213]	Attention/Vigilance (CPT)	**P3b amplitude**	SCZ = 46 HCs = 29 (SCID-DSM-IV) Mean age: SCZ = 25 y; HCs = 25 y	SCZ < HCs No significant correlation between P3b amplitude and attention
Galletly et al., 2007 [214]	Working Memory (Auditory Target Detection Task)	**P3b amplitude**	SCZ = 25 HCs = 25 (SCID-DSM-IV) Mean age: SCZ = 31 y; HCs = 30 y	SCZ < HCs Positive correlation between P3b amplitude and working memory
Heidrich and Strick, 1997 [215]	Verbal intelligence (MWT); Attention (d2 Test)	**P3b amplitude**	SCZ = 13 (DSM-III) Mean age: 31 y	Positive correlation between P3b amplitude and attention
Higuchi et al., 2021 [216]	Working Memory, Speed of Processing, Executive Function, Verbal Memory, Verbal Fluency, Speed of Processing (BACS)	**P3b amplitude** **P3b latency**	ARMS = 33 SCZ = 39 HCs = 28 (CAARMS; ICD-10) Mean age: ARMS = 19 y; SCZ = 24 y; HCs = 22 y	**P3b amplitude** SCZ < ARMS and HCs Positive correlation between P3b amplitude and attention, executive functions and BACS total score. Positive correlation between P300 amplitude and BACS total score **P3b latency** SCZ > ARMS and HCs Negative correlation between P300 latency and BACS total score
Johnston et al., 2005 [210]	Facial emotion recognition task.	**P3b amplitude**	SCZ = 10 HCs = 15 (ICD-10) Mean age: SCZ = 31 y; HCs = 30 y	SCZ = HCs No significant correlation was found between P3b amplitude and emotion recognition
Kayser et al., 1999 [200]	Verbal Memory (task)	**P3b amplitude**	SCZ = 24 HCs = 19 (DSM-IV) Mean age: SCZ = 33 y; HCs = 32 y	SCZ = HCs Positive correlation between P3b amplitude and verbal memory
Kim et al., 2003 [161]	Visual Memory (RCFT); Verbal Fluency (COWA); Executive Functions (WCST and TMT); Verbal Memory and Learning (WAIS)	**P3b amplitude and latency**	SCZ = 22 HCs = 21 (SCID-DSM-IV) Mean age: SCZ = 30 y; HCs = 28 y	SCZ < HCs (amplitude); SCZ = HCs (latency) No significant correlation between P3b and cognitive domains
Kim et al., 2018 [217]	Verbal Learning and (CVLT); Executive Functions (WCST And TMT); Attention (TMT)	**P3b amplitude and inter-trial** **variability**	SCZ = 45 GHR = 32 CHR = 32 HCs = 52 Mean age: SCZ = 26 y; GHR = 25 y; CHR = 21 y; HCs = 24	SCZ, GHR, and CHR < HCs Positive correlation between amplitude and inter-trial stability of P3b and verbal learning
Klein S. D. et al., 2020 [201]	Attention/Vigilance (CPT)	**P3b amplitude**	SCZ = 48 HCs = 68 (DSM-IV) Mean age: SCZ = 46 y; HCs = 45 y	SCZ < HCs Positive correlation between P3b amplitude and attention/vigilance
Kruiper et al., 2019 [211]	Working Memory, Attention, Executive Functions (CANTAB)	**P3b amplitude**	FES = 73 HCs = 93 (ICD-10; DSM-IV) Mean age: FES = 25 y; HCs = 26 y	FES < HCs Positive correlation between P3b amplitude and attention and working memory
Morales-Muñoz et al., 2017 [197]	Attention/Vigilance, Working Memory, Speed of Processing, Verbal Learning, Visual Learning, Reasoning and Problem Solving (MCCB)	**P3b amplitude**	FEP = 38 HCs = 38 (DSM-IV; SCID) Mean age: FEP = 27 y; HCs = 30 y	FEP < HCs Positive correlation between P3b amplitude and attention/vigilance
Nagasawa et al., 1999 [162]	Visual and Verbal Memory and Learning (WMS); Executive Functions (TMT)	**P3b amplitude**	SCZ = 28 HCs = 30 DSM-III Mean age: SCZ = 24 y; HCs = 25 y	SCZ < HCs Positive correlation between P3b amplitude and executive functions
Nieman et al., 2002 [218]	Speed of Processing and Attention (Finger Taping, CPT, TMT, Stroop Test); Intelligence (Four subtests of WAIS); Working Memory (Subjective Ordering Task); Verbal and Visual Memory (Verbal Fluency, CVLT, RCFT)	**P3b amplitude**	SCZ = 45 (DSM IV) HCs = 25 Mean age: SCZ = 21 y; HCs = 23 y	SCZ < HCs Positive correlation between P3b amplitude and attention, verbal learning and memory retrieval
Schreiber et al., 1998 [219]	WAIS; Attention (D2 test of attention)	**P3b amplitude**	HRA = 12 HCs = 12 (DSM-IV) Mean age: HRA = 12 y; HCs = 12 y	HRA < HCs Positive correlation between P3b amplitude and attention
Shajahan et al. 1997 [220]	Attention (WMS), verbal fluency test (WMS), Executive Functions (Stroop), Verbal Learning (CVLT)	**P3b amplitude**	SCZ = 19 HCs = 28 (DSM-IV) Mean age: SCZ = 35 y; HCs = 31 y	SCZ < HCs Positive correlation between P3b amplitude and verbal learning
Schall et al., 1998 [221]	Attention (auditory discrimination task); Executive functions and attention (Stroop task and WCST); Problem solving (TOL); verbal fluency (COWA)	**P3b amplitude**	SCZ treated with clozapine = 15 (SCID; DSM-III) Mean Age: 35 y	Higher baseline values of P3b amplitude were correlated to greater improvements in problem solving and executive functioning assessed at follow-up visit, post-initiation of clozapine treatment. Increases in P3b amplitude (pre-post treatment) were correlated to improvements in an auditory attentive task.
Sumich et al., 2008 [163]	Verbal Memory (WMS-R and RAVLT); Executive Functions (WCST, Verbal fluency, Stroop Colour Word and TMT)	**P3b amplitude and latency**	SCZ = 18 HCs = 18 (DSM-III) Mean age: SCZ = 31 y; HCs = 28 y	SCZ < HCs Positive correlation between P3a amplitude and executive functions, delayed visual memory
**N400**				
Boudewyn et al., 2017 [222]	Vocabulary (NDVT); Working Memory (Listening Span); Attention/Vigilance (CPT)	**N400 amplitude**	SCZ = 26 HCs = 23 (DSM-IV; SCID) Mean age: SCZ = 23 y; HCs = 22 y	Altered N400 sensitivity to context in SCZ compared to HCs. Negative correlation between N400 amplitude and vocabulary task
Favre et al., 2020 [196]	Executive function, Speed of Processing, Verbal and Visual Memory (Cogstate/DRM Paradigm)	**N400 amplitude**	SCZ = 25 HCs = 24 (ICD-10) Mean age: SCZ = 23 y; HCs = 22 y	SCZ < HCs No significant correlation between N400 amplitude and cognitive domains
Green et al., 2017 [160]	Episodic memory (Memory Task Performance)	**N400 amplitude**	SCZ = 24 HCs = 19 (MINI) Mean age: 37 y	SCZ > HCs No significant correlation between N400 amplitude and episodic memory
Jackson et al., 2014 [223]	Verbal Learning and Verbal Memory (CVLT)	**N400 amplitude**	SCZ = 41 PSY = 48 HCs = 35 (DSM-IV; SCID) Mean age: SCZ = 45; PSY = 43; HCs = 39	SCZ and OP > HCs Negative correlation between N400 amplitude and verbal learning and memory
Lepock et al., 2021 [224]	Attention/Vigilance, Working Memory, Speed of Processing, Verbal Learning, Visual Learning, Reasoning and Problem Solving; neurocognitive composite score (MCCB)	**N400 amplitude**	CHR = 35 (SIPS) Mean age: 21 y	Negative correlation between N400 amplitude and neurocognitive composite score
**ERN and Pe**				
Alain et al., 2002 [225]	Attention and Cognitive Control (Stroop Task)	**ERN amplitude**	SCZ = 12 HCs = 12 (DSM-IV; SCID) Mean age: SCZ = 31 y; HCs = 32 y	SCZ = HCs No significant correlation between ERN amplitude and attention and cognitive control
Chan et al., 2015 [226]	Error awareness (Accuracy scores subjectively assigned during a Flanker task)	**ERN amplitude** **Pe amplitude**	PSY = 14 HCs = 12 (DSM-IV: SCID-I) Mean age: SCZ = 37 y; HCs = 37 y	**ERN amplitude** PSY > HCs No correlation between ERN amplitude and error awareness **Pe amplitude** PSY < HCs Positive correlation between Pe amplitude and self-awareness of mistakes
Foti et al., 2020 [227]	Executive functions (TMT, Stroop, LNS); Attention/Speed of Processing (TMT); General cognitive abilities (WAIS)	**ERN amplitude** **Pe amplitude**	PSY = 181 (93 with SCZ) HCs = 242 (DSM-IV; SCID) Mean age: PSY = 48 y; HCs = 51 y	**ERN amplitude** PSY > HCs Negative correlation between ERN amplitude and executive functions, attention and general cognitive ability **Pe amplitude** PSY < HCs Positive correlation between ERN amplitude and executive functions and attention
Francisco et al., 2020 [205]	Executive Functions (D-KEFS)	**ERN amplitude** **Pe amplitude**	22q11.2 DS = 27 SCZ = 15 HCs (matched to 22q11.2 DS) = 27 HCs (matched to SCZ) = 15 Mean age: 22q11.2 DS and matched HCs = 22 y; SCZ and matched HCs = 43 y	**ERN amplitude** 22q11.2 DS and SCZ > HCs Negative correlation between ERN amplitude and executive functions **Pe amplitude** 22q11.2 DS and SCZ < HCs Positive correlation between P300 amplitude and executive functions

The column “EEG-Indices” reports the EEG index considered in that study, while in the last column, differences between patients and controls, as well as correlations with cognition for that measure are reported. Participants diagnosed with DiGeorge syndrome (22q11.2DS); Auditory Consonant Trigram Test (ACT); German version of the Auditory Verbal Learning Test (AVLT); Japanese version of the Brief assessment of cognitive function of schizophrenia (BACS-J); Comprehensive Assessment of at-risk Mental States (CAARMS); the Cambridge Neuropsychological Test Automated Battery (CANTAB); Category Fluency (CFT); subjects at clinical high-risk for psychosis (CHR); Controlled Oral Word Association Test (COWA); Continuous Performance Test (CPT); Comprehensive Test of Phonological Processing (CTOPP); the California Verbal Learning Test (CVLT); Clozapine Group (CLZ); The Free Inquiry section of the Delis-Kaplan Executive Function System (D-KEFS); Deese–Roediger–McDermott (DRM); Delayed response task (DRT); Digit Span Backward Test (DS-BT); Digit Span Forward Test (DS-FT); Emotional Faces Recognition (ERT) tests; subjects with first-episode schizophrenia (FES); Facial Expressions of Emotion-Stimuli and Test (FEEST); Figural Memory Test (FMT); the Finger-Tapping Test (FTT); subjects at genetic-high-risk for psychosis (GHR); Gray Oral Reading Test (GORT-4); healthy controls (HCs); High-risk adolescents (HRA); Japanese Verbal Learning Test (JVLT); International Statistical Classification of Diseases and Related Health Probles (ICD-10); Intra/Extra Dimensional Set Shift (IED); Identical Pairs version (IP); the Luria–Nebraska neuropsychological battery (LNNB); Letter-Number Span Test (LNS); the Mehrfachwahl-Woertschatz Test (MWT); Mental Control (MC); Consensus Cognitive Battery (MCCB); Multiple Choice Vocabulary Test (MWT); Mini International Neuropsychiatric Inventory (MINI) assessment; Mini-Mental State Exam (MMSE); pitch mismatch-negativity (pMMN); duration P3 (dP3); double (pitch and duration) mismatch-negativity (dblMMN); the Multiple Word Recognition Test (MWT-B); National Adult Reading Test (NART); the Nelson-Denny vocabulary test (NDVT); One Touch Stockings of Cambridge (OTS); Paired Associates Learning (PAL); PENN Neurocognitive Battery (PENN CNB); Profile of Nonverbal Sensitivity (PONS); individuals with a history of psychosis or psychotic disorder (PSY); the Rey Auditory Verbal Learning Test (RAVLT); Repeatable Battery for the Assessment of Neuropsychological Status (RBANS); Rivermead Behavioural Memory Test (RBMT); The Rey–Osterrieth Complex Figure Test (RCFT); Rapid Visual Processing (RVP); Structured Clinical Interview for DSM-IV (SCID); Subjects with schizophrenia (SCZ); Structured Interview for Psychosis-Risk Syndromes (SIPS); Sustained potential (SP); Spatial Working Memory (SWM); treatment as usual (TAT); targeted cognitive training (TCT); Japanese Verbal Learning Test (JVLT); the Trail-Making Test (TMT); Tower of London (TOL); Patients maintained on typical neuroleptics (TYP); The Wechsler Adult Intelligence Scale (WAIS); Wechsler Abbreviated Scale of Intelligence (WASI); the Wisconsin Card Sorting Test (WCST); he Wechsler Intelligence Scale for Children (WISC-V); the Wechsler Logical Memory (Prose Recall) Test (WLMPR); Wechsler Memory Scale (WMS); Word Fluency Test (WFT); Wide Range Achievement Test-3 (WRAT-3); Wechsler Test of Adult Reading (WTAR).

**Table 4:** Sleep Studies.

Study	Cognitive Domains	EEG Indices	Sample Size	Correlations between EEG Indices and Cognitive Functions in Patients and High-Risk Subjects
Baandrup et al., 2019 [260]	Verbal memory, Working memory, Verbal fluency, Attention, Processing speed and Executive functions (BACS)	**Sleep spindles** **characteristics**	SCZ = 37 (ICD-10) Mean age: 47 y	No significant correlation between sleep spindle characteristics and cognitive domains
Bartsch et al., 2019 [261]	Memory consolidation (MST)	**Slow waves density amplitude and** **duration and sleep spindles** **coordination**	SCZ = 21 HCs = 19 (SCID-DSM-IV) Mean age: SCZ = 34; HCs = 36)	SCZ = HCs (for slow wave density, amplitude and duration); SCZ < HCs (coupling of slow waves and sleep spindles) No correlation between amplitude, density and duration of slow waves or sleep spindles features and memory consolidation
Forest et al., 2007 [262]	Attention (attention task)	**Sleep spindle** **density and sleep stages duration**	SCZ = 8 HCs = 8 (SCID-DSM-IV) Mean age: SCZ = 31 y; HCs = 21 y	SCZ = HCs Positive correlation between sleep spindles density and percentage of time spent in stage 4 of sleep and attention
Gerstenberg et al., 2020 [263]	Verbal Learning (AVLT); Executive functioning (TMT)	**Sleep spindle** **density**	EO-SCZ = 12 HCs = 24 (DSM-IV; SCID) Mean age: EO-SCZ = 17 y; HCs = 17 y	SCZ < HCs No significant correlation between sleep spindle density and verbal learning or executive functioning
Göder et al., 2015 [264]	Memory/Recognition (Memory task)	**Sleep spindle** **density**	SCZ = 16 HCs = 16 (ICD-10) Mean age: SCZ = 29 y; HCs = 28 y	SCZ < HCs Positive correlation between sleep spindle density and memory performance
Keshavan et al., 2004 [265]	Memory (WMS); Executive functions (WCST)	**Non-linearity EEG scores during sleep**	SCZ = 10 HCs = 10 (SCID-DSM-IV) Mean age: SCZ = 20 y; HCs = 20 y	SCZ < HCs Positive correlation between non-linear EEG complexity and memory and executive functions
Manoach et al., 2014 [266]	Executive functions (WCST and TMT); Verbal learning (CVLT)	**Sleep spindle amplitude and density**	SCZ = 17 HCs = 25 (DSM-IV; SCID) Mean age: SCZ = 28 y; HCs = 27 y	SCZ < HCs Positive correlation between sleep spindle density and amplitude and executive functions
Ramakrishnan et al., 2012 [267]	Problem solving (ToL, Digit Symbol Test); Executive functioning (TMT)	**K-complexes during sleep**	SCZ = 20 (DSM-IV; SCID) Mean age: 41 y	Positive correlation between the number of K-complexes and executive functioning and problem solving
Wamsley et al., 2012 [268]	Motor memory (motor memory task)	**Sleep spindle** **number and density**	SCZ = 21 HCs = 17 (DSM-IV; SCID) Mean age: SCZ = 36 y; HCs = 34 y	SCZ < HCs Positive correlation between sleep spindle density and memory performance

The column “EEG Indices” reports the EEG index considered in that study, while in the last column, differences between patients and controls, as well as correlations with cognition for that measure are reported. Auditory Verbal Learning Test (AVLT); the California Verbal Learning Test (CVLT); Controlled Oral Word Association Test (COWAT); subjects with early onset schizophrenia (EO-SCZ); Healthy controls (HCs); International Statistical Classification of Diseases and Related Health Probles (ICD); motor-sequencing task (MST); subjects with schizophrenia (SCZ); Tower of London (ToL); Wisconsin Card Sorting Test (WCST); Wechsler Memory Scale (WMS); Trail-Making Test (TMT).

## Data Availability

The data presented in the current review are all available within the article.
